# Supplementary figures and images for: A dynamic calcium-force relationship model for sag behavior in fast skeletal muscle
Source: PLoS Comput Biol. 2023 Jun 8;19(6):e1011178. doi: 10.1371/journal.pcbi.1011178 (PMC10284414; doi:10.1371/journal.pcbi.1011178)

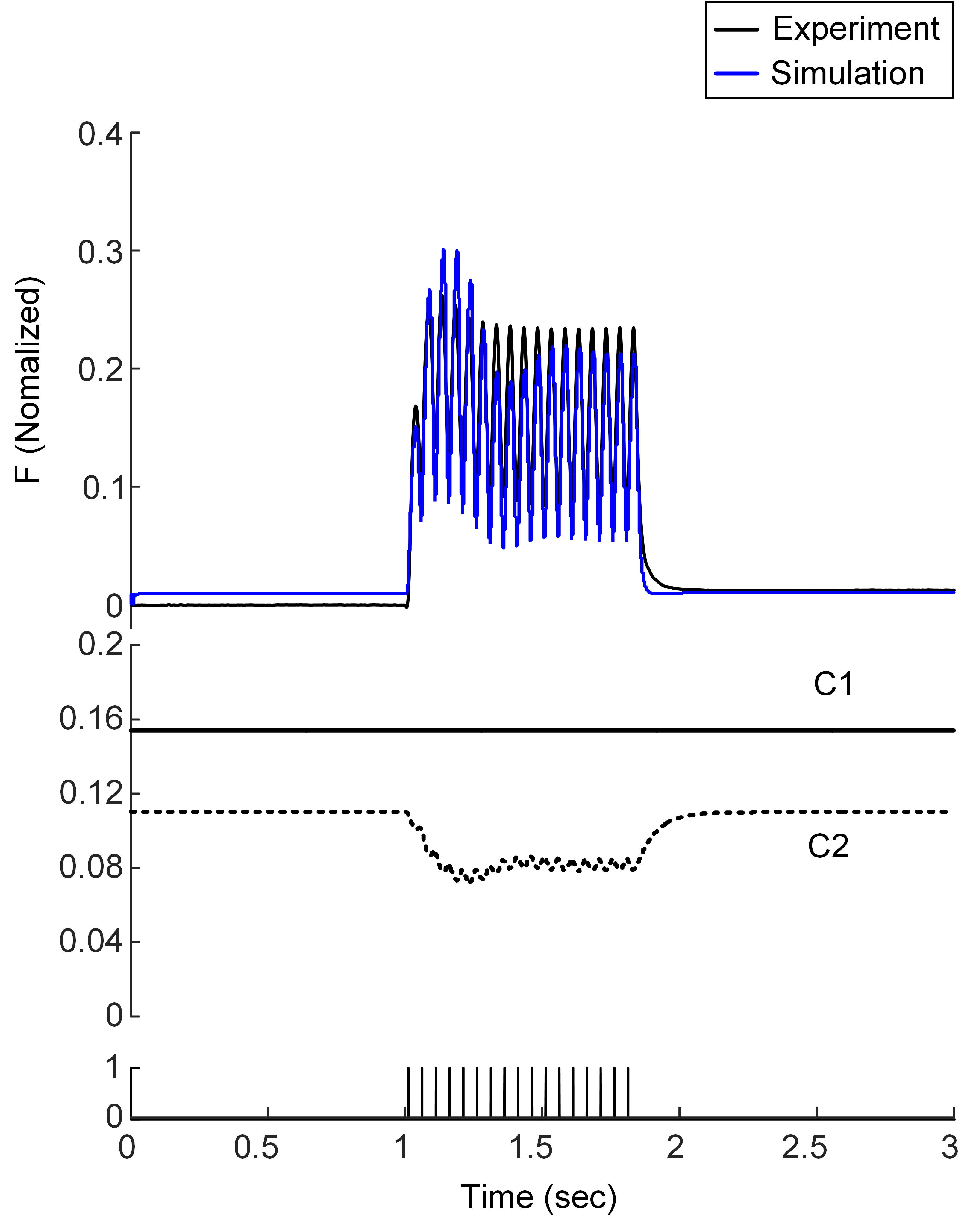

Supplement: S1 Fig — Unfused tetanus (upper), change in C2 (middle) and current stimulation (20 Hz, bottom). Black and blue lines indicate the data obtained from the experiment and simulation. (TIF) [file pcbi.1011178.s001.tif]

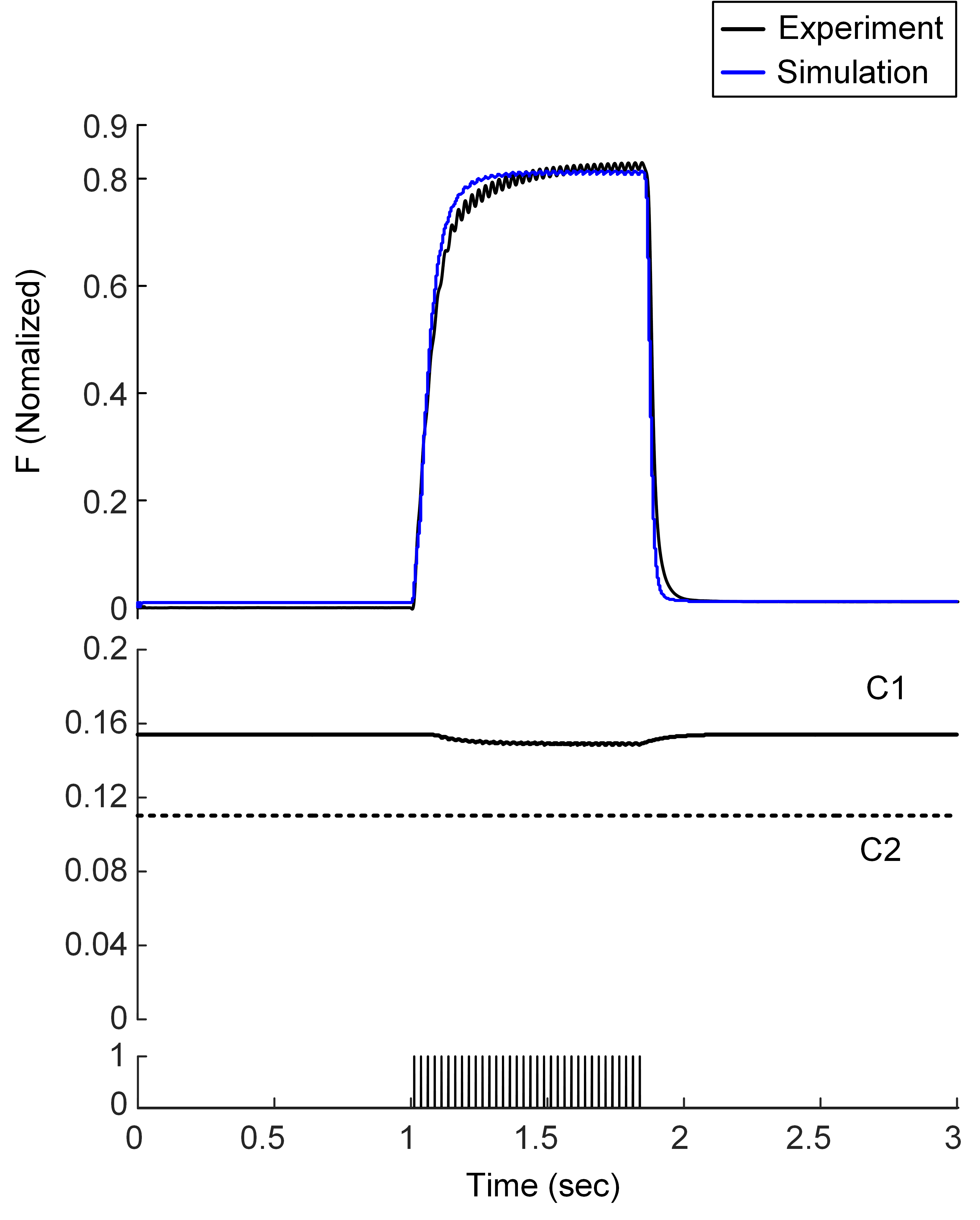

Supplement: S2 Fig — Unfused tetanus (upper), change in C1 (middle) and current stimulation (40 Hz, bottom). Black and blue lines indicate the data obtained from the experiment and simulation. (TIF) [file pcbi.1011178.s002.tif]

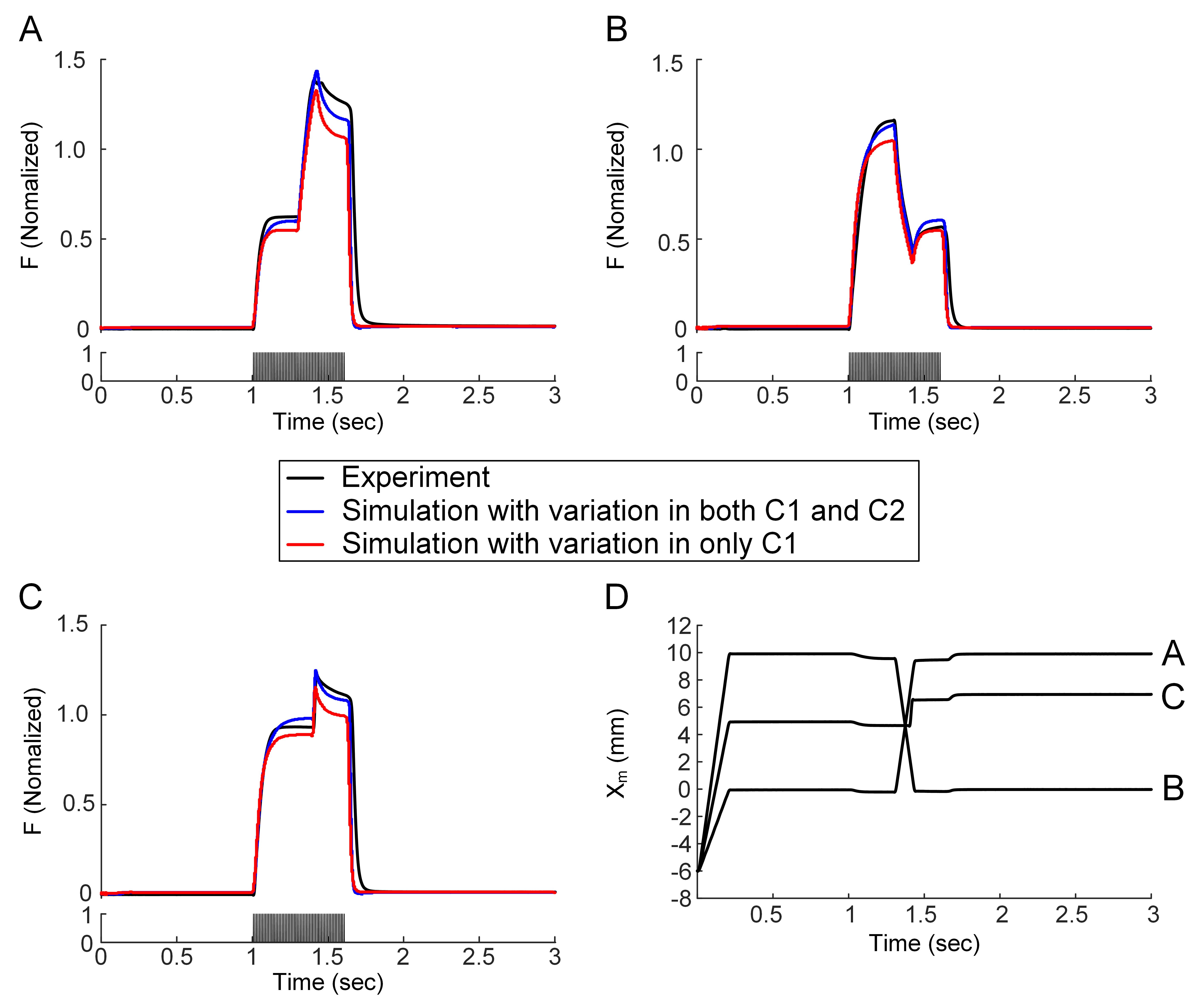

Supplement: S3 Fig — A-C. Force production (upper) at current stimulation (100 Hz, bottom) during lengthening, shortening, and step lengthening of the muscle-tendon length, respectively. D. Profiles of the muscle-tendon length (Xm) variation for A, B, and C. Black, blue and red lines in A-C indicate the experiment, simulation with and without (C2n1 = 0) the slope steepening of the calcium-force relationship, respectively. (TIF) [file pcbi.1011178.s003.tif]

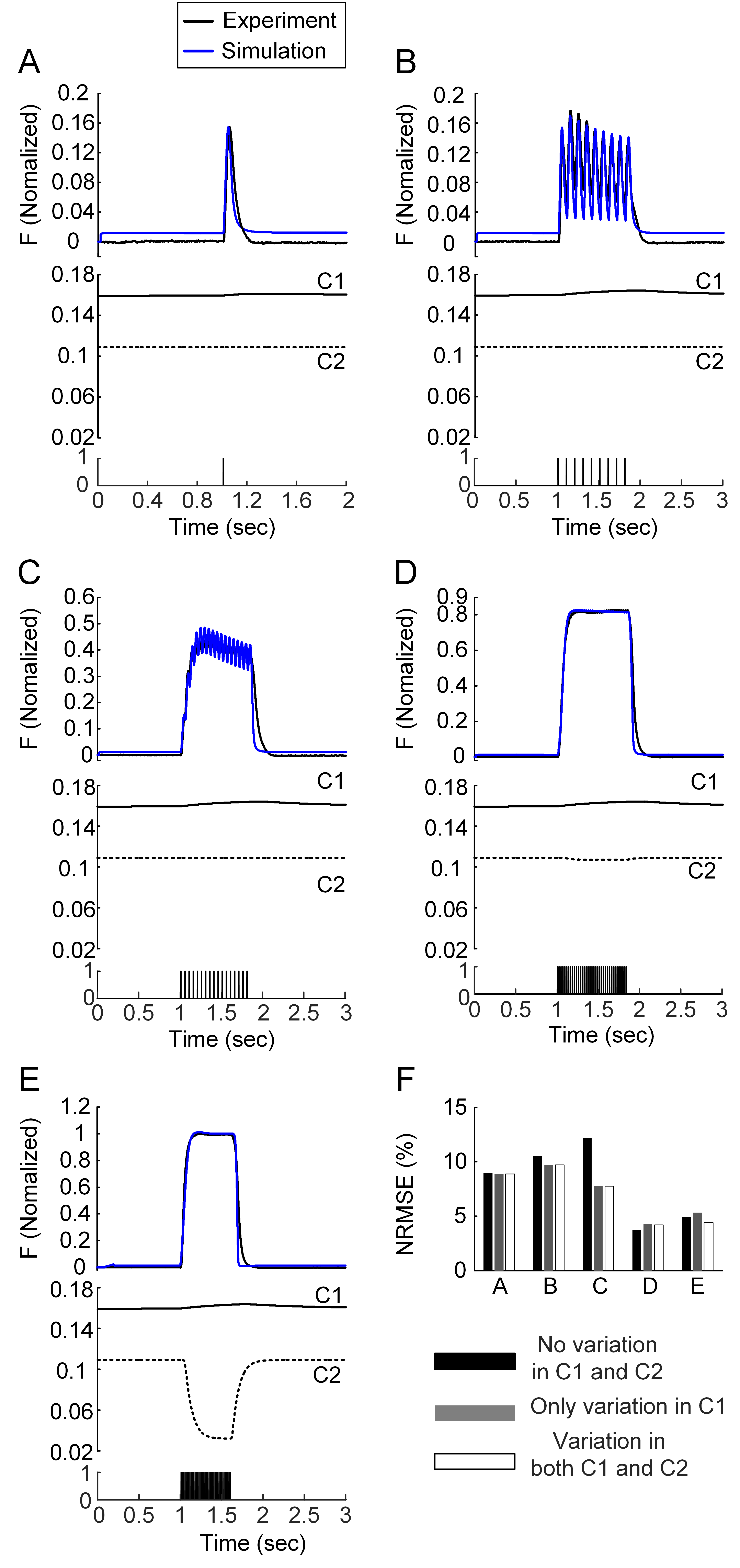

Supplement: S4 Fig — A. Twitch (upper), change in C1 & C2 (middle) and current stimulation (bottom). B. Unfused tetanus (upper), change in C1 & C2 (middle) and current stimulation (10 Hz, bottom). C. Unfused tetanus (upper), change in C1 & C2 (middle) and current stimulation (20 Hz, bottom). D. Unfused tetanus (upper), change in C1 & C2 (middle) and current stimulation (40 Hz, bottom). E. Fused tetanus (upper), change in C1 & C2 (middle) and current stimulation (100 Hz, bottom). F. Simulation error with no variation in C1 & C2 (black), only variation in C1 (gray) and variation in both C1 & C2 (white) at the stimulation frequency of 1 Hz (A), 10 Hz (B), 20 Hz (C), 40 Hz (D) and 100 Hz (E). Black and blue lines in A-E indicate the data obtained from the experiment and simulation with variation in both C1 & C2. (TIF) [file pcbi.1011178.s004.tif]

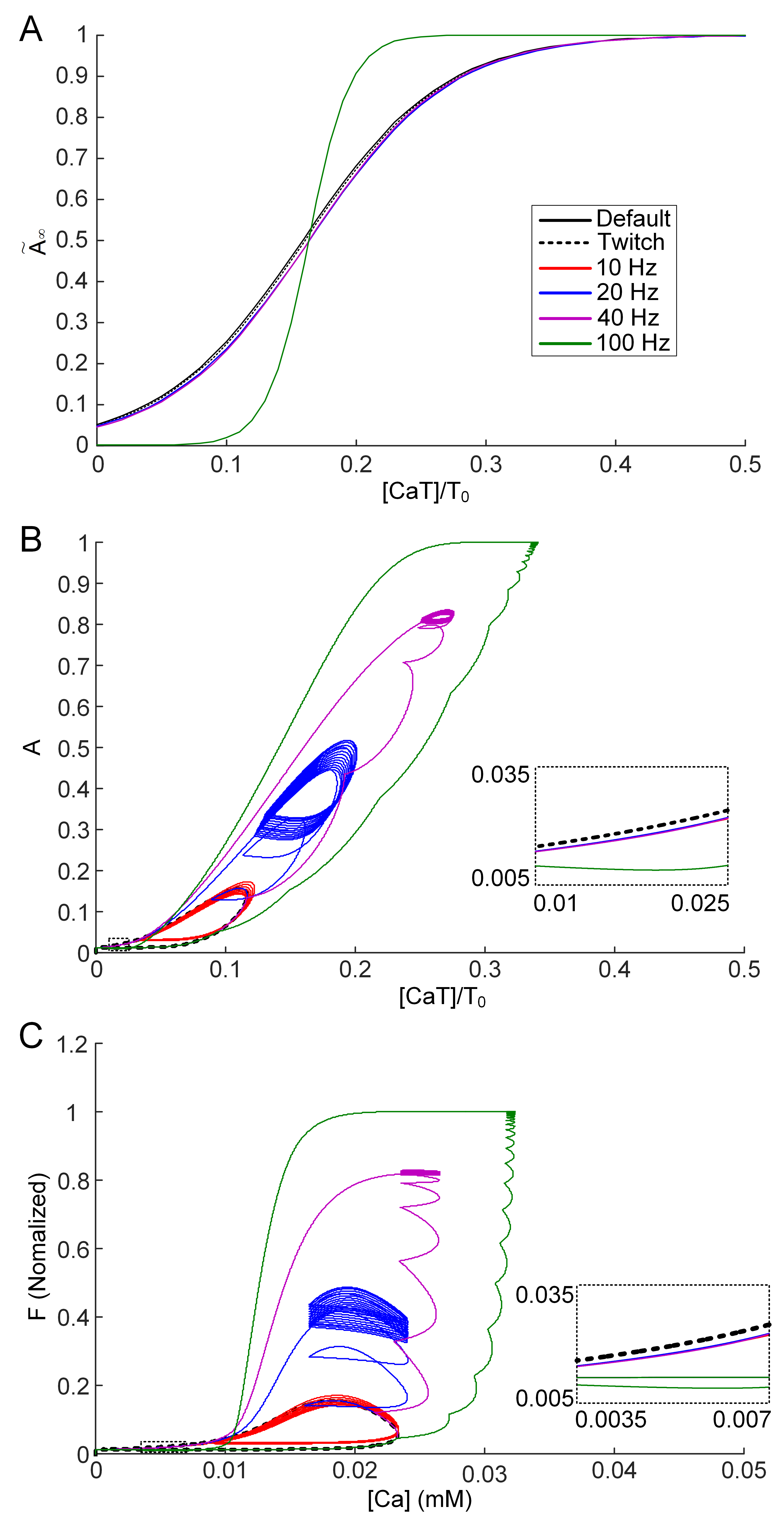

Supplement: S5 Fig — A. Steady-state relationship of muscle activation (A˜∞) to calcium binding troponin relative to the total troponin concentration (CaT/T0) in the initial and maximally varied states at various stimulation frequencies. B. Transient relationship of muscle activation (A) to CaT/T0 at various levels of stimulation frequency. C. Transient relationship of muscle force (F) and sarcoplasmic calcium (Ca) at various levels of stimulation frequency. Insets indicate the transient relationship between calcium and activation (B) and calcium and force (C) on the relaxation phase of force production. (TIF) [file pcbi.1011178.s005.tif]

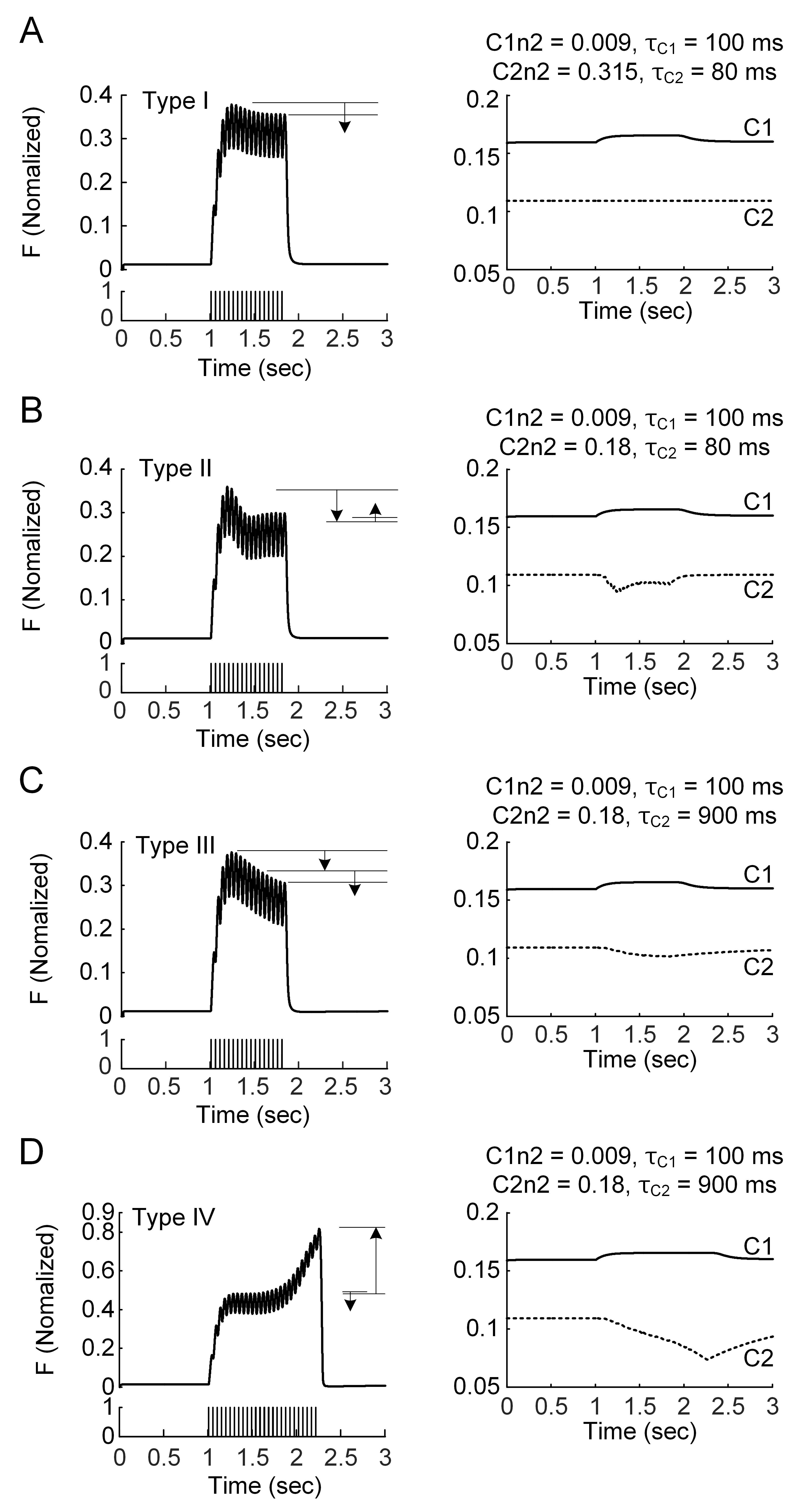

Supplement: S6 Fig — A. Simple sag form at 20 Hz current stimulation (left) and changes in C1 and C2 (right) with default values of C2n2 and τC2. B-D. Complex sag forms at 20 Hz (B and C) and 30 Hz (D) stimulation frequencies (left) and changes in C1 and C2 (right) with variations in C2n2 and τC2. Arrows indicate the direction of force production after the initial peak force. (TIF) [file pcbi.1011178.s006.tif]

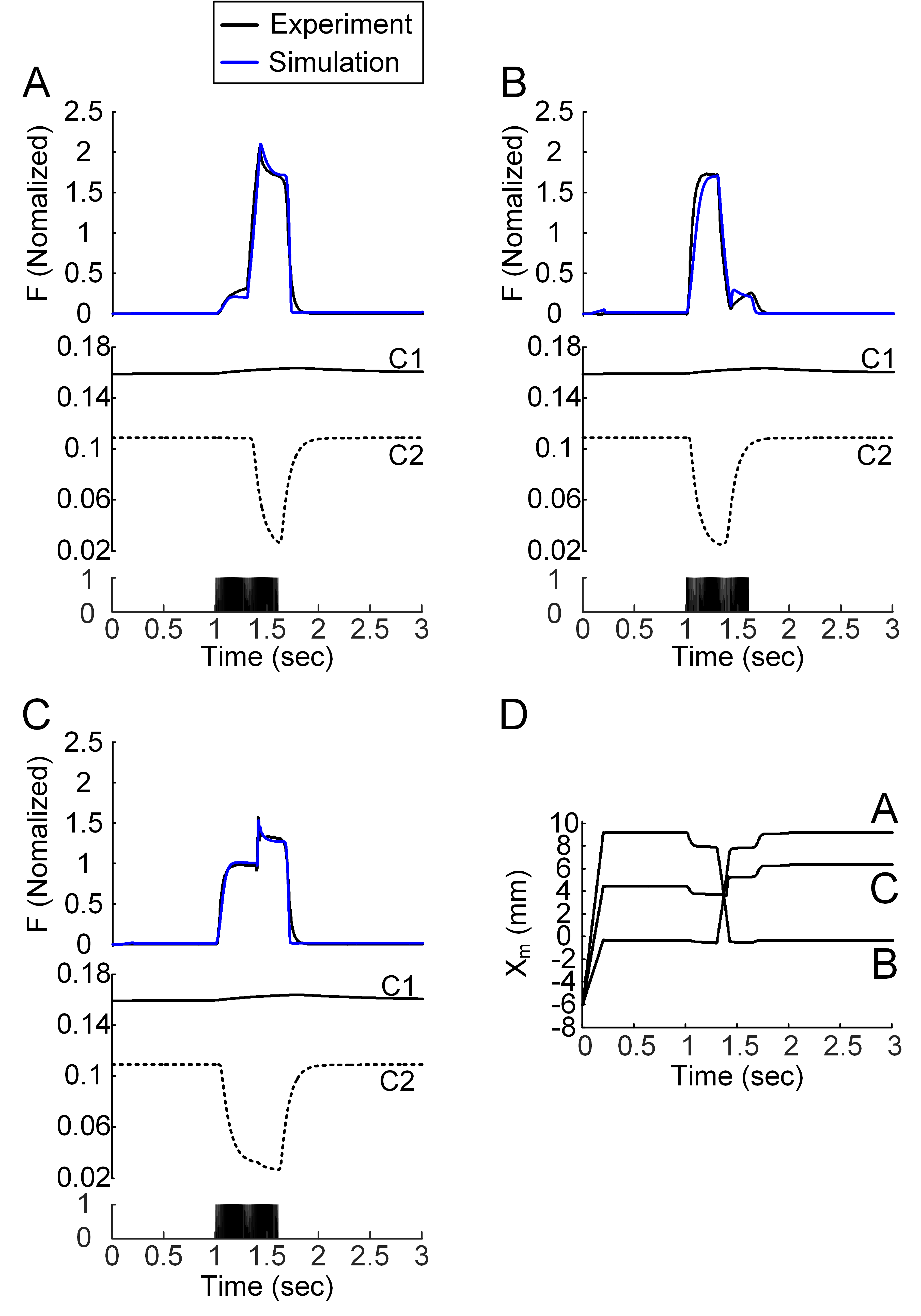

Supplement: S7 Fig — A. Force responses during lengthening (upper), changes in C1 and C2 (middle) and current stimulation (100 Hz, bottom). B. Force responses during shortening (upper), changes in C1 and C2 (middle) and current stimulation (100 Hz, bottom). C. Force responses during step lengthening (upper), changes in C1 and C2 (middle) and current stimulation (100 Hz, bottom). D. Profiles of the muscle-tendon length variation for A, B and C. Black and blue lines indicate the experimental and simulated data. (TIF) [file pcbi.1011178.s007.tif]

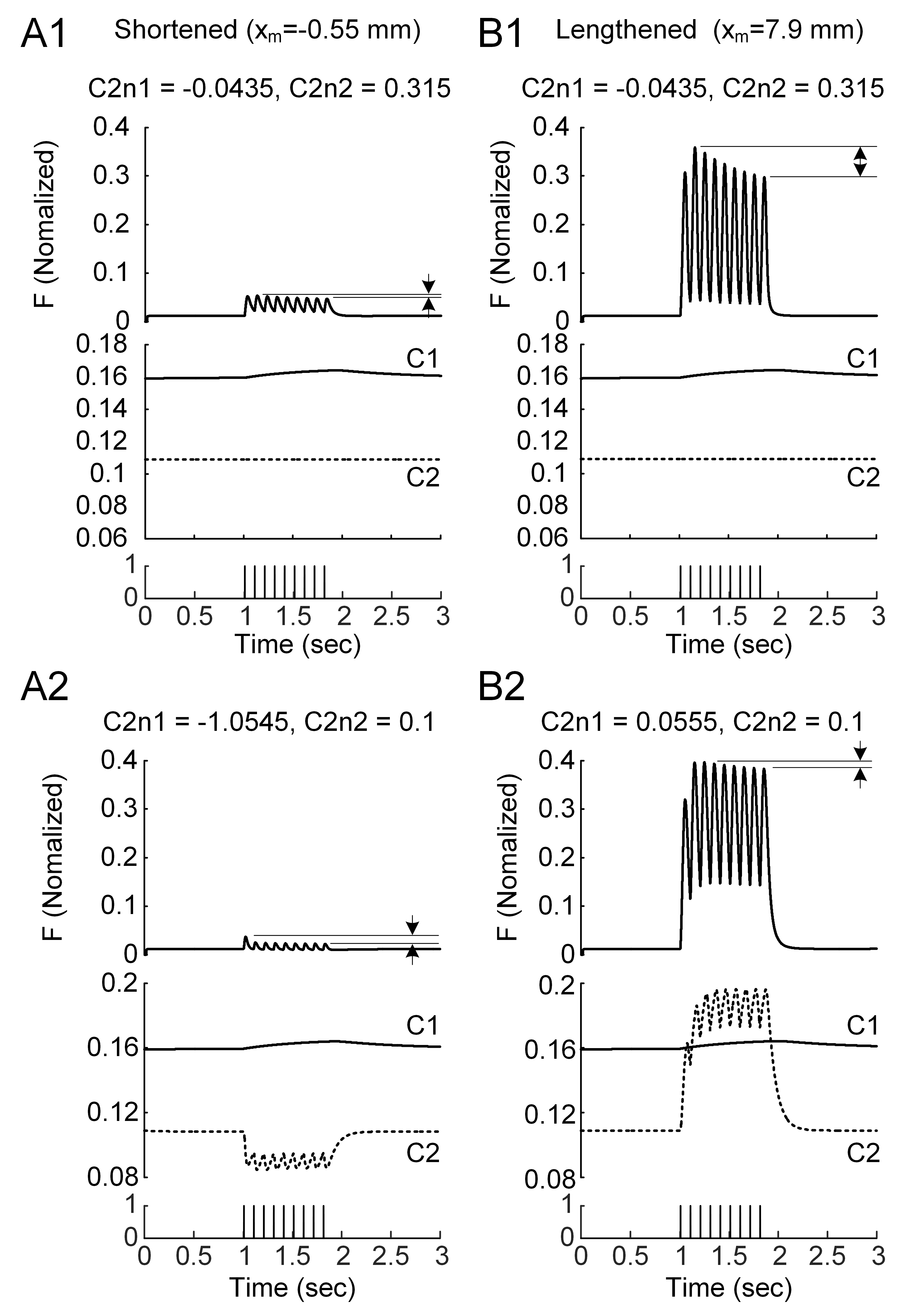

Supplement: S8 Fig — A1 & B1. Unfused tetanus (upper), changes in C1 and C2 (middle) and 20 Hz current stimulation (bottom) with no change in the slope of the calcium-force relationship at the physiologically minimal (-0.55 mm) and maximal (7.9 mm) muscle-tendon length. A2 & B2. Unfused tetanus (upper), changes in C1 and C2 (middle) and 20 Hz current stimulation (bottom) with slope variation in the calcium-force relationship at the physiologically minimal and maximal muscle-tendon length. Arrows indicate the degree of force decline after the initial peak force. (TIF) [file pcbi.1011178.s008.tif]

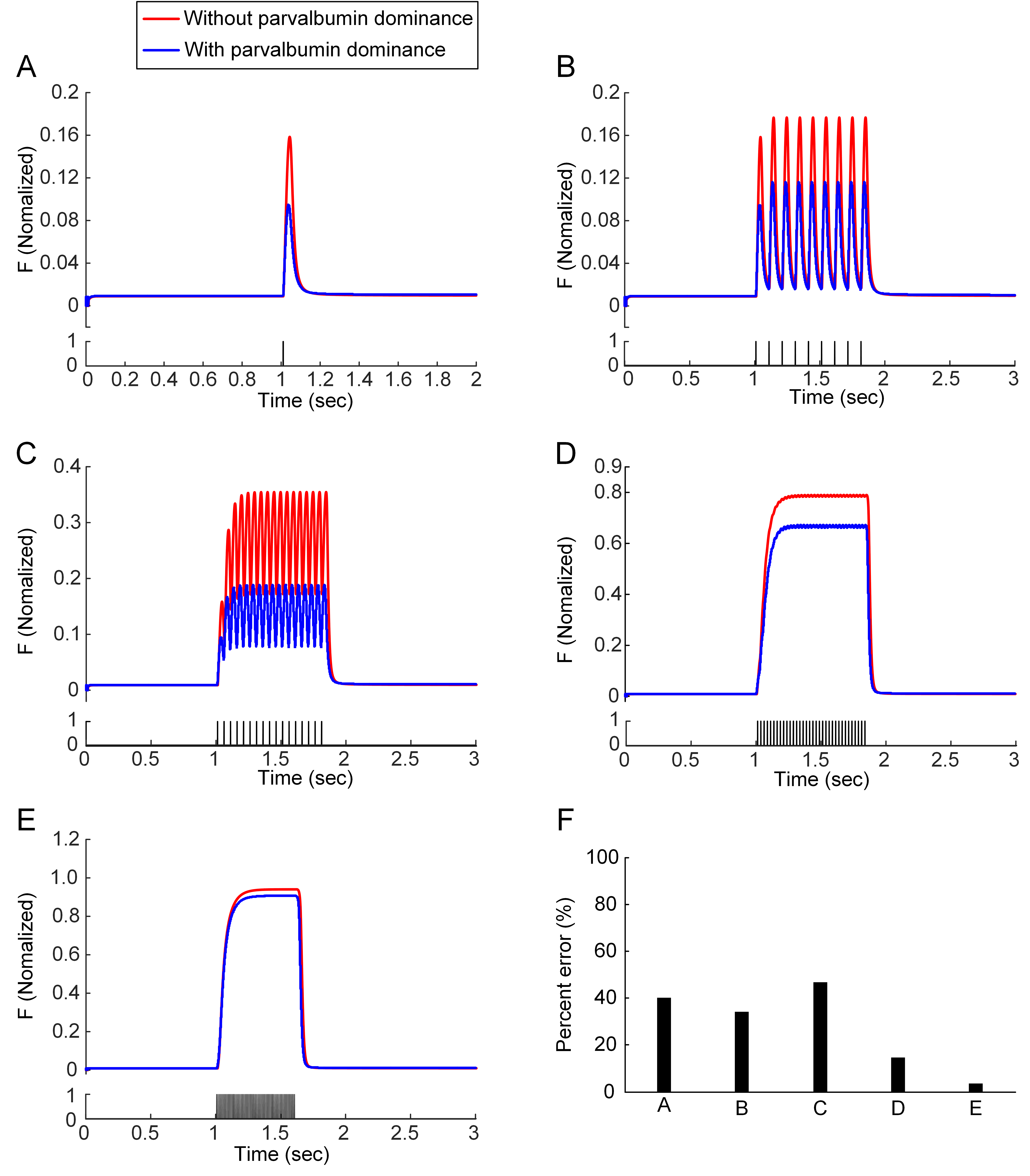

Supplement: S9 Fig — A-E. Forces (upper) and current stimulation (bottom) of 1, 10, 20, 40, and 100 Hz, respectively. Blue and red lines indicate the simulation with parvalbumin dominance and that presented in Fig 2. F. Percent error of peak force produced with the parvalbumin dominance relative to the canonical case in Fig 2 for A-E. (TIF) [file pcbi.1011178.s009.tif]

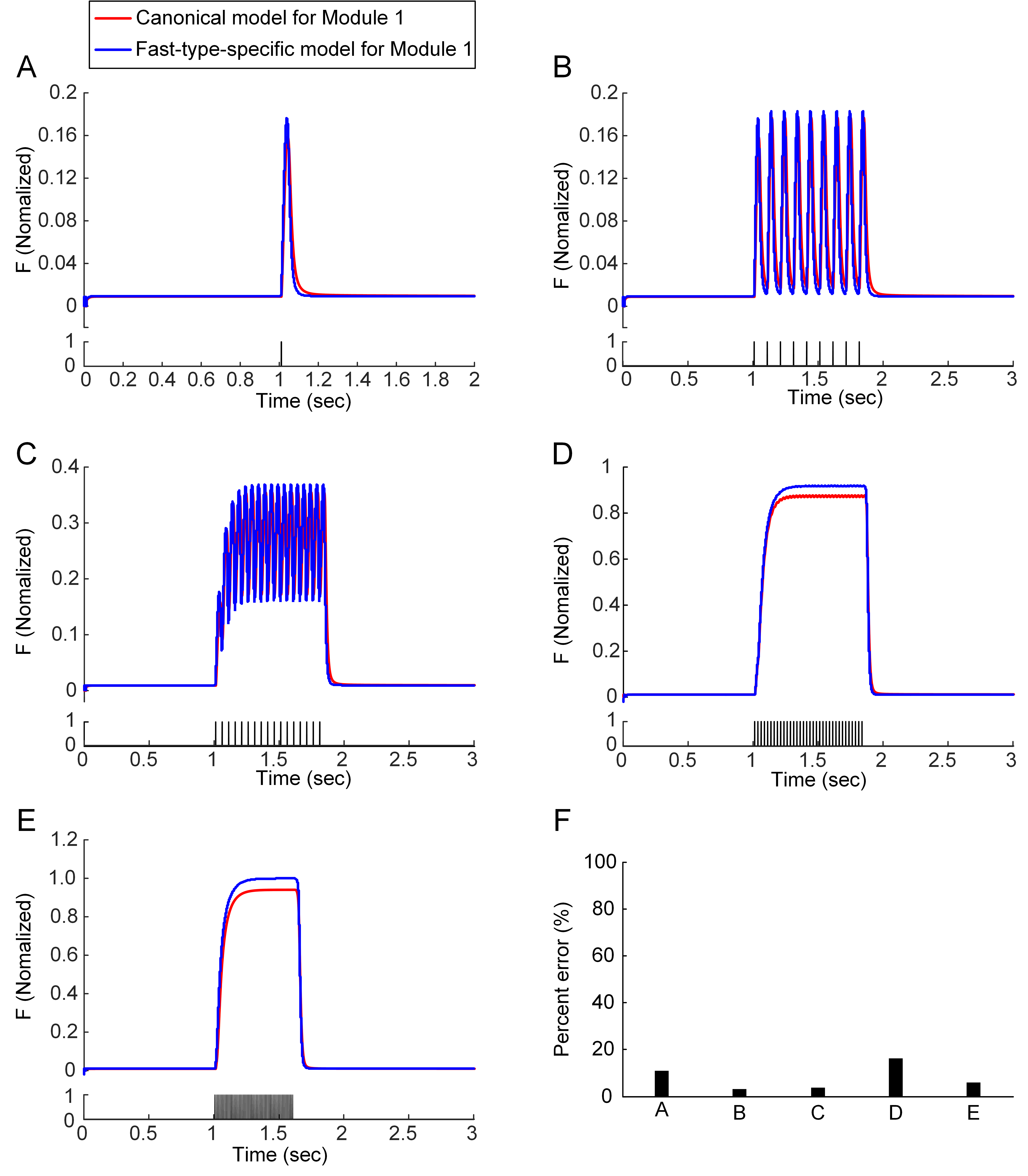

Supplement: S10 Fig — A-E. Forces (upper) and current stimulation (bottom) of 1, 10, 20, 40, and 100 Hz, respectively. Blue and red lines indicate the simulation with two regulatory sites of troponin and that presented in Fig 2. F. Percent error of peak force produced with two regulatory sites of troponin relative to the canonical case in Fig 2 for A-E. (TIF) [file pcbi.1011178.s010.tif]

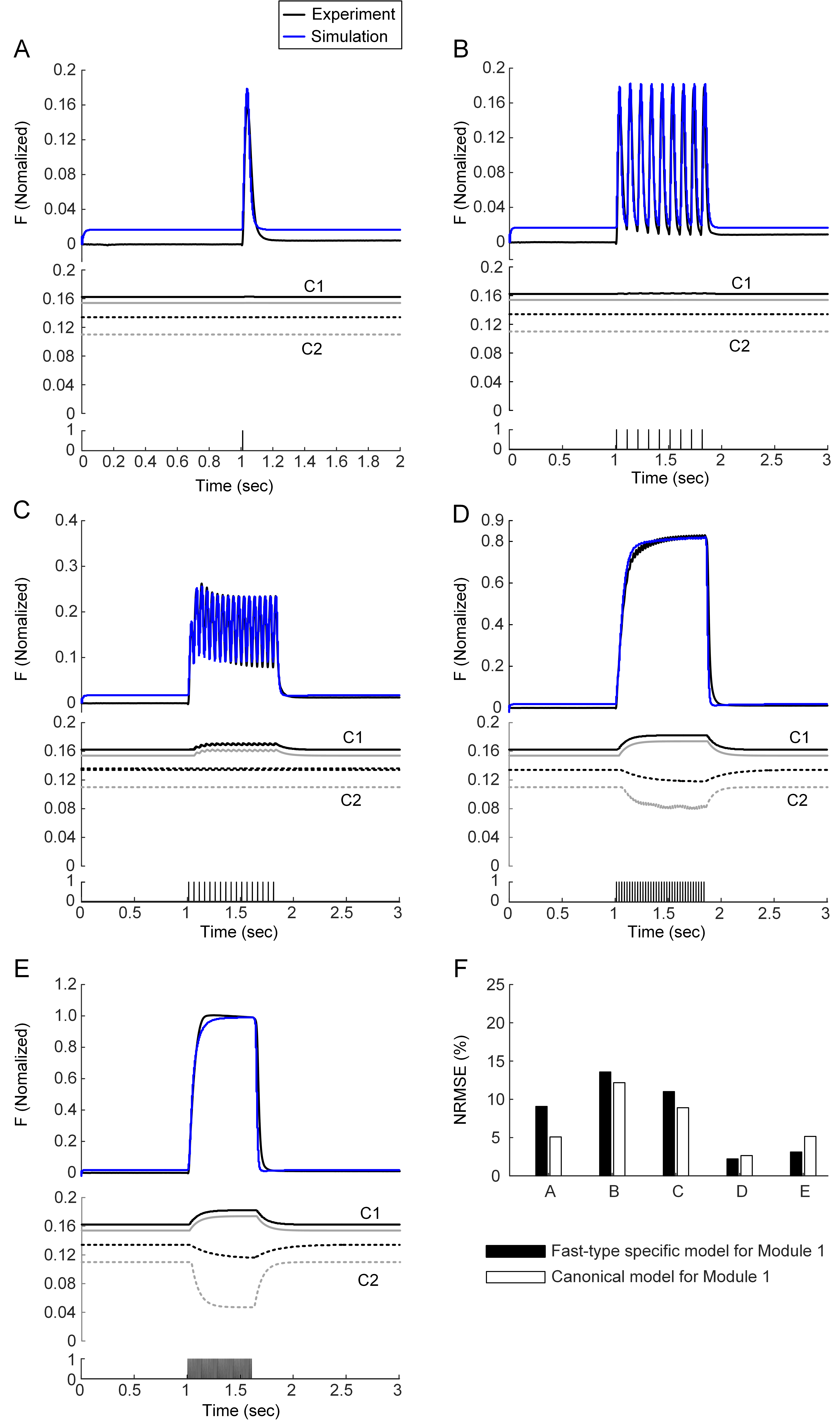

Supplement: S11 Fig — A-E. Forces (upper), change in C1 & C2 (middle), and current stimulation (bottom) of 1, 10, 20, 40, and 100 Hz, respectively. Black and blue lines in A-E indicate the experimental and simulated force data for CAT14. Gray lines indicate the changes in C1 & C2 of the muscle-tendon model with the canonical model for Module 1 in Fig 4. F. Normalized root mean square error (NRMSE) between the experiment and simulation with the fast-type-specific model for Module 1 (black) in the Panels A-E. NRMSE for the canonical model of Module 1 (white) presented in Fig 4 was overlapped for the purpose of comparison. (TIF) [file pcbi.1011178.s011.tif]

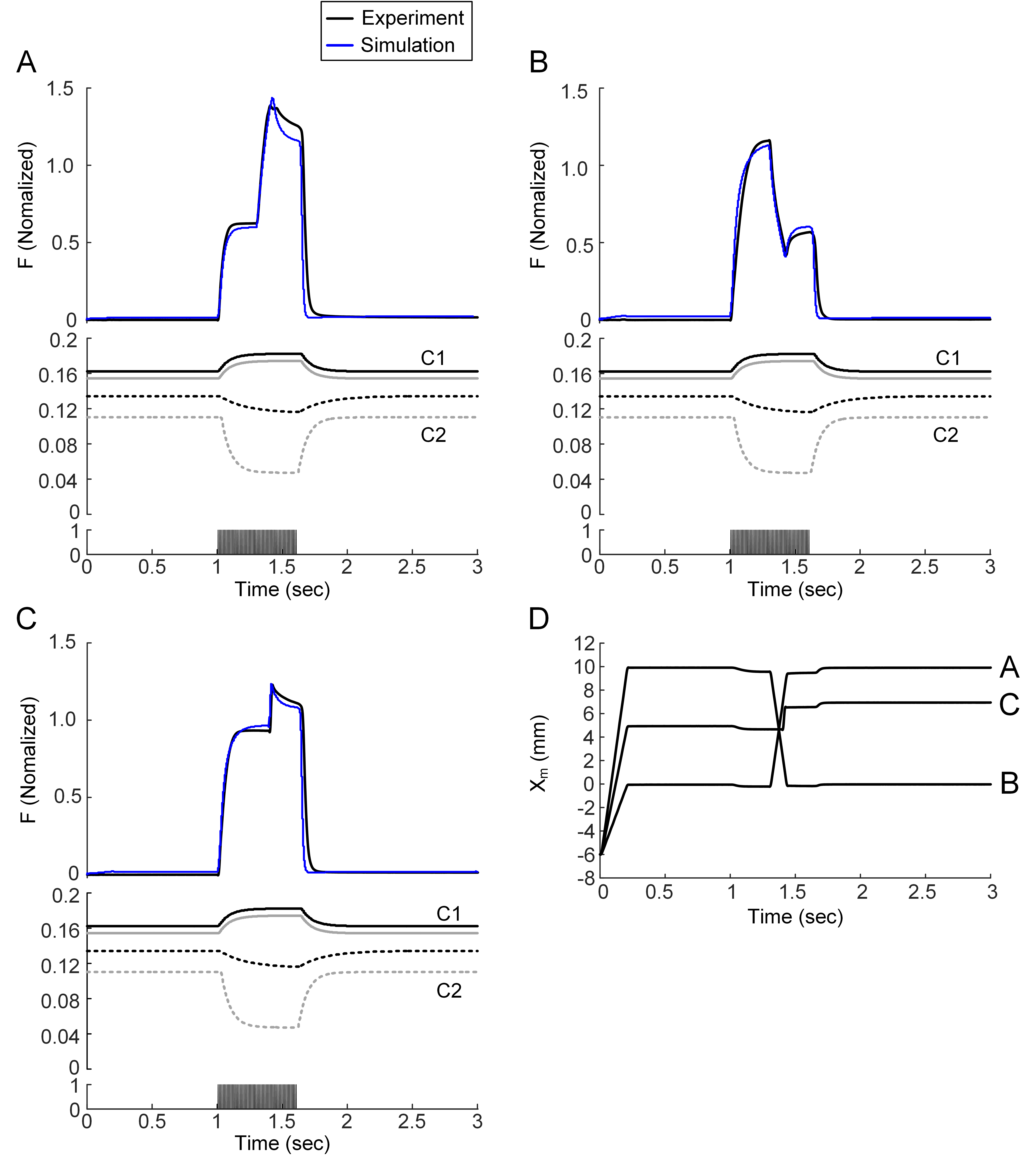

Supplement: S12 Fig — A-C. Force production (upper) at current stimulation (100 Hz, bottom) during lengthening, shortening, and step lengthening of the muscle-tendon length, respectively. Black and blue lines in A-C indicate the experimental and simulated force data for CAT14. Gray lines indicate the changes in C1 & C2 of the muscle-tendon model with the canonical model for Module 1 in Fig 8. D. Profiles of the muscle-tendon length (Xm) variation for A, B, and C. (TIF) [file pcbi.1011178.s012.tif]

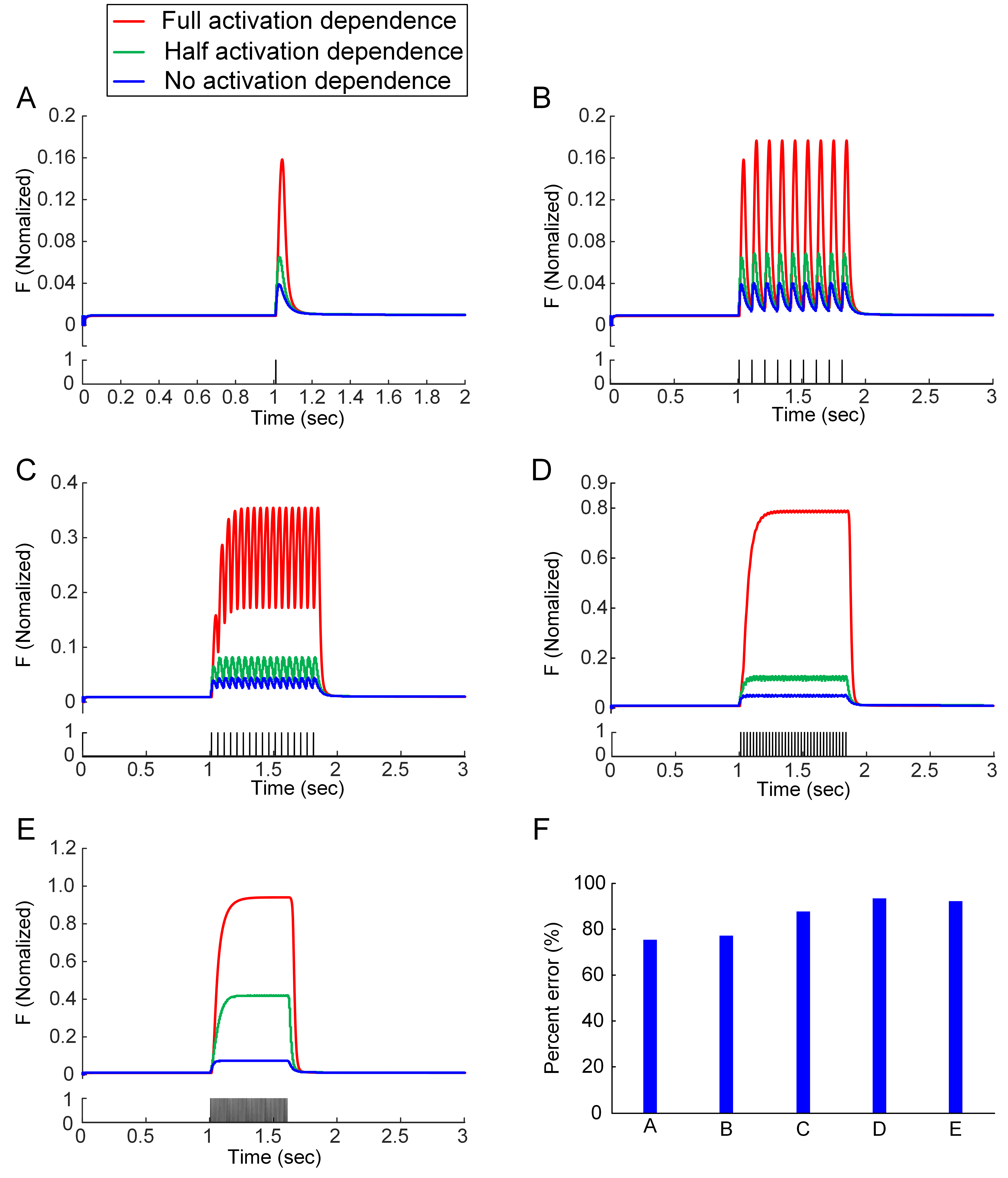

Supplement: S14 Fig — A-E. Predicted forces (upper) and current stimulation (bottom) of 1, 10, 20, 40, and 100 Hz, respectively. F. Percent error between peak force with and without the activation dependence of K6 in A-E. Red, green, and blue colors indicate the full, half, and no activation dependence in K6. (TIF) [file pcbi.1011178.s014.tif]

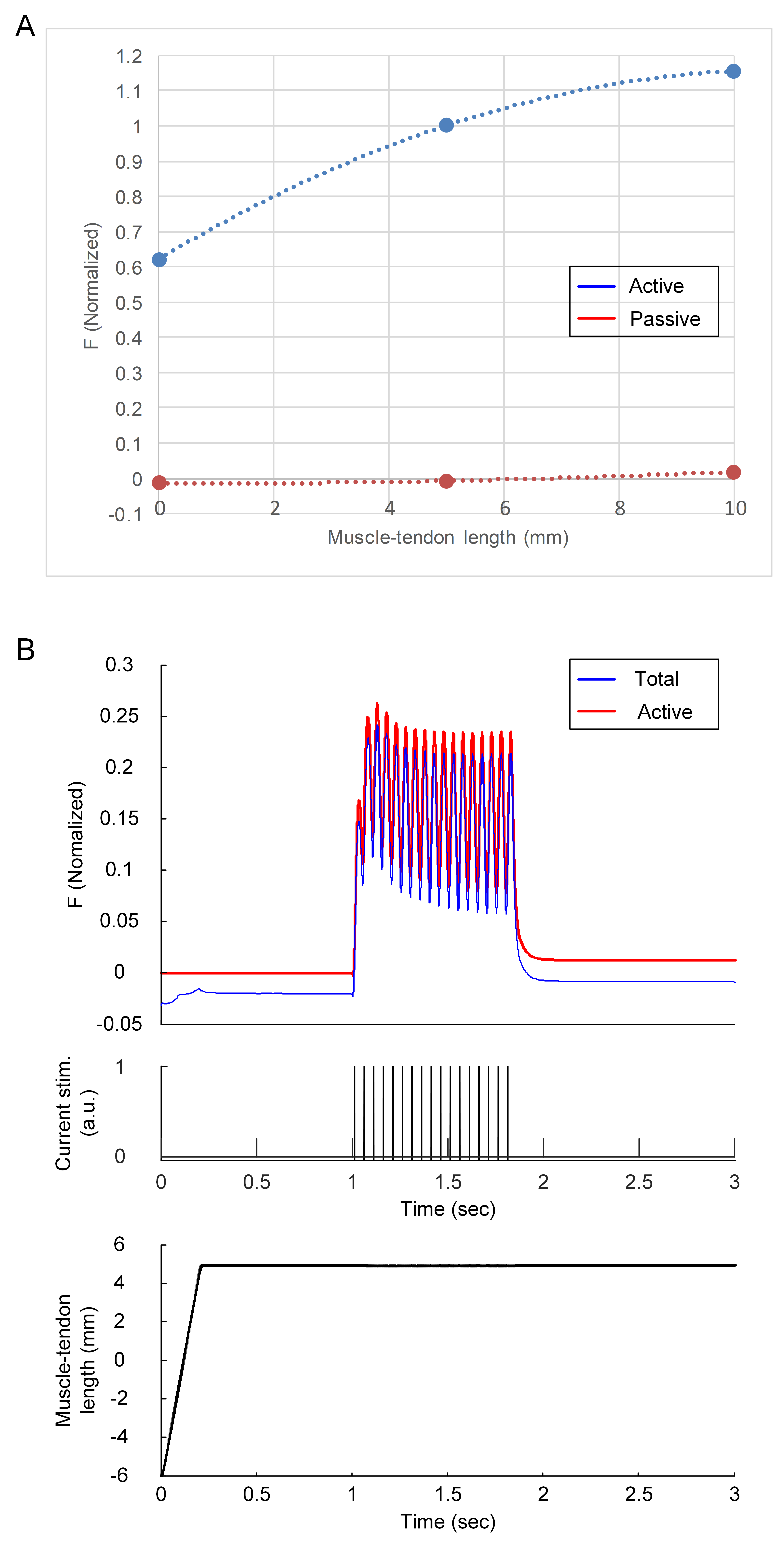

Supplement: S15 Fig — A. Active and passive force-length relationship with (blue) and without (red) current stimulation (100 Hz) for CAT14. B. Normalized force responses (upper) to current stimulation (20 Hz, middle) at the intermediate length (Xm = 5 mm, bottom) for CAT14. Blue and red lines indicate the total force produced by the whole muscle and its active force calculated by subtracting the passive force measured without current stimulation from the total force. (TIF) [file pcbi.1011178.s015.tif]
