# Supplementary material for: A dynamic calcium-force relationship model for sag behavior in fast skeletal muscle
Source: PLoS Comput Biol. 2023 Jun 8;19(6):e1011178. doi: 10.1371/journal.pcbi.1011178 (PMC10284414; doi:10.1371/journal.pcbi.1011178)
Supplement: Attachment — Submitted filename: PCOMPBIOL-D-22-01287-review-notes.pdf [file pcbi.1011178.s018.pdf]

PLOS Computational Biology

Modular modeling framework reveals dynamic alterations in calcium sensitivity during force production in skeletal muscle

--Manuscript Draft--

**Manuscript Number:**	PCOMPBIOL-D-22-01287
**Full Title:**	Modular modeling framework reveals dynamic alterations in calcium sensitivity during force production in skeletal muscle
**Short Title:**	Dynamic calcium sensitivity of muscle force
**Article Type:**	Research Article
**Keywords:**	skeletal muscle; calcium-force relation; calcium sensitivity; crossbridge formation; sag behavior
**Abstract:**	In-vitro studies using isolated or skinned muscle fibers suggest that the sigmoidal relationship between intracellular calcium concentration and force production may depend upon muscle type and activity. The goal of this study was to investigate whether and how the calcium-force relation changes during force production under physiological conditions of muscle excitation and length in fast skeletal muscles. A computational framework was developed to identify the dynamic variation in the calcium-force relation during force generation over a full physiological range of stimulation frequencies and muscle lengths in cat gastrocnemius muscles. Unlike in slow muscles such as soleus, the calcium concentration for the half-maximal force needed to drift rightward to reproduce the progressive force decline over time or sag behavior observed during unfused isometric contractions at the intermediate length under low frequency stimulation (i.e., 20 Hz). The slope at the calcium concentration for the half-maximal force was required to drift upward for force enhancement during unfused isometric contractions at the intermediate length under high frequency stimulation (i.e., 40 Hz). The slope variation in the calcium-force relation played a crucial role in shaping the sag behavior across different muscle lengths. The muscle model with dynamic variations in the calcium-force relation also accounted for the length- and velocity-force properties measured under full excitation. These results imply that the calcium sensitivity of force-inducing crossbridge formation between actin and myosin filaments may be operationally altered in accordance with the mode of neural excitation and muscle movement in intact fast muscles.
**Additional Information:**	
**Question**	**Response**
**Financial Disclosure**<br><br>Enter a financial disclosure statement that describes the sources of funding for the work included in this submission. Review the submission guidelines for detailed requirements. View published research articles from *PLOS Computational Biology* for specific examples.<br><br>This statement is required for submission and **will appear in the published article** if the submission is accepted. Please make sure it is accurate.	HK received funding from the National Research Foundation of Korea (NRF, https://www.nrf.re.kr) (2021060027) and the Ministry of Science and ICT (MSIT, https://www.msit.go.kr) (DGIST R&D Program 21-BT-06) and CH received funding from the National Institutes of Health (NIH, https://www.nih.gov) (NS071951 and NS62200). The funders had no role in study design, data collection and analysis, decision to publish, or preparation of the manuscript.

Powered by Editorial Manager® and ProduXion Manager® from Aries Systems Corporation

**Unfunded studies**<br>Enter: *The author(s) received no specific funding for this work.*<br><br>**Funded studies**<br>Enter a statement with the following details:<br>• Initials of the authors who received each award<br>• Grant numbers awarded to each author<br>• The full name of each funder<br>• URL of each funder website<br>• Did the sponsors or funders play any role in the study design, data collection and analysis, decision to publish, or preparation of the manuscript?<br>• **NO** - Include this sentence at the end of your statement: *The funders had no role in study design, data collection and analysis, decision to publish, or preparation of the manuscript.*<br>• **YES** - Specify the role(s) played.<br><br>* typeset	
**Competing Interests**<br><br>Use the instructions below to enter a competing interest statement for this submission. On behalf of all authors, disclose any competing interests that could be perceived to bias this work—acknowledging all financial support and any other relevant financial or non-financial competing interests.<br><br>This statement **will appear in the published article** if the submission is accepted. Please make sure it is accurate. View published research articles from *PLOS Computational Biology* for specific examples.	NO authors have competing interests.

**NO authors have competing interests**<br><br>Enter: *The authors have declared that no competing interests exist.*<br><br>**Authors with competing interests**<br><br>Enter competing interest details beginning with this statement:<br><br>*I have read the journal's policy and the authors of this manuscript have the following competing interests: [insert competing interests here]*<br><br>* typeset<br><br>This statement is required for submission and **will appear in the published article** if the submission is accepted. Please make sure it is accurate and that any funding sources listed in your Funding Information later in the submission form are also declared in your Financial Disclosure statement.	
**Data and Code Availability**<br><br>From the time of publication, Authors are required to make fully available and without restriction all data and computational code underlying their findings. Please see our PLOS Data Policy page for detailed policy information, and our Code Sharing page for specific information on code sharing.<br><br>A **Data Availability Statement**, detailing where the data (and code, if applicable) can be accessed, is required at first submission. Insert your Data Availability Statement in the box below. The statement you provide **will be published in the article**, if accepted.<br><br>Please see the Data Reporting section of our submission guidelines for instructions on what you need to include in your Data Availability Statement.	All relevant data are within the manuscript and its Supporting Information files.

If the data and code are all contained in your submission files, please state: *All relevant data are within the manuscript and its Supporting Information files.*<br><br>**PLOS allows rare exemptions to address legal and ethical concerns.** If you have legal or ethical restrictions, please detail these in your Data Availability Statement below for the Journal team to consider.	

Powered by Editorial Manager® and ProduXion Manager® from Aries Systems Corporation

Manuscript Click here to access/download;Manuscript;ms_ploscomt_f8.docx

Modular modeling framework reveals dynamic alterations in calcium sensitivity during force production in skeletal muscle

Running title: dynamic calcium sensitivity of muscle force

Authors:

[1,2]Hojeong Kim and [3,4,5]CJ Heckman

Affiliations:

[1] Division of Biotechnology, Institute of Convergence Research, DGIST, Daegu, Republic of Korea

[2] Department of Interdisciplinary Studies, DGIST, Daegu, Republic of Korea

[3] Department of Neuroscience, Feinberg School of Medicine, Northwestern University, Chicago, IL, United States

[4] Physical Therapy and Human Movement Sciences, Feinberg School of Medicine, Northwestern University, Chicago, IL, United States

[5] Physical Medicine and Rehabilitation, Feinberg School of Medicine, Northwestern University, Chicago, IL, United States

Corresponding author:

Hojeong Kim

Hojeong.kim03@gmail.com

Keywords: skeletal muscle, calcium-force relation, calcium sensitivity, crossbridge formation, sag behavior

Abstract

In-vitro studies using isolated or skinned muscle fibers suggest that the sigmoidal relationship between intracellular calcium concentration and force production may depend upon muscle type and activity. The goal of this study was to investigate whether and how the calcium-force relation changes during force production under physiological conditions of muscle excitation and length in fast skeletal muscles. A computational framework was developed to identify the dynamic variation in the calcium-force relation during force generation over a full physiological range of stimulation frequencies and muscle lengths in cat gastrocnemius muscles. Unlike in slow muscles such as soleus, the calcium concentration for the half-maximal force needed to drift rightward to reproduce the progressive force decline over time or sag behavior observed during unfused isometric contractions at the intermediate length under low frequency stimulation (i.e., 20 Hz). The slope at the calcium concentration for the half-maximal force was required to drift upward for force enhancement during unfused isometric contractions at the intermediate length under high frequency stimulation (i.e., 40 Hz). The slope variation in the calcium-force relation played a crucial role in shaping the sag behavior across different muscle lengths. The muscle model with dynamic variations in the calcium-force relation also accounted for the length- and velocity-force properties measured under full excitation. These results imply that the calcium sensitivity of force-inducing crossbridge formation between actin and myosin filaments may be operationally altered in accordance with the mode of neural excitation and muscle movement in intact fast muscles.

Author summary

Intracellular calcium concentration is a crucial factor determining the magnitude of active force in skeletal muscle. The relationship between muscle force and intracellular calcium concentration has been characterized by observing force responses of isolated or skinned muscle fibers at different calcium concentration levels controlled under in-vitro conditions. However, whether and how the calcium-force relation changes under

physiological conditions, including neural excitation and muscle movement, remains unclear. Because of current experimental limitations, we developed a computational framework that allows us to identify the calcium-force relation for force generation over a full physiological range of stimulation frequencies and muscle lengths in cat medial gastrocnemius muscles. The results indicate that, unlike in slow muscles such as soleus, dynamic variation in the calcium-force relation is required to reproduce the complex force profiles observed in fast skeletal muscles under physiological conditions. This study may provide insights into the dynamic properties of calcium-mediated crossbridge formation between thin and thick filaments for force generation and a computational framework for realistic muscle modeling.

Main Text

Introduction

Skeletal muscles are specialized tissues that can contract to generate force in response to neural signals from spinal motoneurons. It is commonly thought that contractile forces are induced from crossbridge formation between actin and myosin filaments in the sarcomere (1-4), which is activated by calcium release from the sarcoplasmic reticulum according to neural excitation (5). Thus, the transfer function of force inducing crossbridge formation across different excitation levels has been characterized by measuring steady-state force responses at various constant calcium concentration levels (6).

Isolated or skinned muscle fiber experiments on the calcium sensitivity of muscle force have shown larger force production at higher calcium concentrations compared to the lower constant calcium concentration levels at steady state (7). This sigmoid steady-state calcium-force relation implies the cooperativity and saturation of crossbridge formation as the level of muscle excitation increases (8-10). The steady-state calcium-force relation has also been shown to be specific to the type of muscle fibers (slow-twitch versus fast-twitch in (11)) in mammals (12-16) and humans (17). Recent in vitro studies have further demonstrated that the shape of the calcium-force relation can be considerably altered by inorganic phosphate molecules known to accumulate during force production (18) or by variation in muscle length (7). However, it remains elusive whether and how the calcium-force relation varies in intact muscle fibers under physiological conditions of muscle excitation and length.

Computational studies have suggested that the calcium-force relation might differ depending on the type of muscle fibers (slow-twitch versus fast-twitch) under physiological conditions. Slow-twitch muscles have been shown to produce a force whose peak is maintained during isometric contractions at any level of stimulation frequency (19). These isometric forces could be reproduced for a full

physiological range of stimulation frequencies with no changes in the calcium-force relation in computational models (20). In contrast, fast-twitch muscles have been shown to produce a force that declines rapidly after its peak during isometric contractions under unfused stimulation conditions (21). The impaired crossbridge formation by the inhibitory effects of inorganic phosphate accumulation has been computationally demonstrated as a potential mechanism for this rapid force decline during continuous submaximal contractions (22). The decline of free calcium ion concentration in the sarcoplasm by the reduction in calcium release from the sarcoplasmic reticulum (23) and the accelerated uptake of calcium into the sarcoplasmic reticulum (24) has been proposed to be involved in the final phase of muscle fatigue showing the accelerated force reduction in several seconds after the onset of continuous maximal contraction (23, 25).

Here, we focus on the dynamic variation in the calcium-force relation during force production in fast skeletal muscles prior to the final phase of muscle fatigue. It is hypothesized that both the midpoint and the slope of the sigmoid calcium-force relation dynamically change for force production under physiological conditions. Because of current experimental limitations, a computational approach is developed to test our hypothesis. The comparison between experiment and simulation results shows that the dynamic variation in the calcium-force relation is required to replicate the force responses of cat medial gastrocnemii over the full physiological range of stimulation frequencies and muscle lengths. This study provides insights into the dynamic properties of calcium-sensitive crossbridge formation under physiological conditions and a computational framework for realistic modeling of complex muscle behaviors.

Materials and Methods

In-situ experiments

The properties of average active force production by single sarcomeres were characterized by measuring the active force of the whole muscle in decerebrated cats (3). All procedures were approved by the Animal Care Committee at Northwestern University. Details of the surgical preparations and experimental protocols have been previously reported (26). Briefly, adult cats (CAT14 for model development and CAT12 for model validation) were anesthetized, the medial gastrocnemius (MG) was exposed, and the distal tendon was attached to a computer-controlled puller. All distal hindlimb nerves except the medial gastrocnemius nerve were disconnected, only ipsilateral dorsal roots from L4 to S2 were transected to eliminate sensory feedback from the medial gastrocnemius, and decerebration was performed at the midbrain. Hindlimb and core temperatures were maintained within physiological limits. Electrical impulses with a width of 0.1 ms were applied to control muscle force either using stainless steel wires in the proximal and distal portions of the muscle belly or via hook electrodes on the ventral roots. A supra axial current (50-100% above that required to elicit full recruitment) was used to produce repeatable and consistent forces during all trials. Length of muscle-tendon unit was controlled by a position servo system and measured by a linear variable differential transformer attached to the puller shaft. Muscle forces were measured by a strain gage-based transducer in series with the shaft. Raw data for muscle force and length were smoothed using the function ("smooth" with options of "0.01" for span and "loess" for method) built in MATLAB software (MathWorks, Natick, MA). To isolate the active force response, the force data measured without the stimulation (i.e., passive response) were subtracted from those measured with the stimulation (i.e., active response) under the same condition of muscle-tendon length. The passive and active trials were separated by a 30 s rest period, and all active trials were also separated by at least one minute to minimize fatigue.

Input protocols

The calcium-force relation was dynamically evaluated during twitch, unfused and fused tetani produced with constant stimulation frequencies (i.e., 1, 10, 20, 40 and 100 Hz) at the intermediate length ($X_{m,0.5}$, 5 mm for CAT14 and 3.64 mm for CAT12) between the physiologically minimal ($X_{m,0}$, 0 mm for CAT14 and -0.55 mm for CAT12) and maximal ($X_{m,1}$, 10 mm for CAT14 and 7.9 mm for CAT12) length that have been observed during locomotor-like movements in cats (27-29). The representative types of sag behavior were reproduced under constant stimulation frequencies of 20 and 30 Hz at $X_{m,0.5}$ for both CAT14 and CAT12. The length and velocity dependence of the dynamics of the calcium-force relation was investigated during shortening and lengthening at constant velocities and step lengthening over a very short period of time under full excitation (100 Hz) for both CAT14 and CAT12.

Modular modeling framework

A biophysical model of muscle-tendon complex experimentally validated for the adult cat soleus (predominantly slow twitch fibers (19)) in in-situ conditions was adapted to replicate the force production of the adult cat medial gastrocnemius (predominantly fast twitch fibers (30)). The derivation and description of model equations for the model of slow skeletal muscles has been fully presented in a previous study (20). Briefly, the canonical model consists of three modules to capture the essential processes that muscle fibers undergo for force production (Figure 1). The first module (i.e., Module 1) transforms the electrical signals coming from either spinal motoneurons or electrical stimulation into the concentration of free calcium (Ca) within the sarcoplasm (SP). The concentration of sarcoplasmic calcium was determined by multiple factors, including the reaction of calcium (Ca) and calsequestrin (CS) in the sarcoplasmic reticulum (SR), calcium release (R) and uptake (U) through the membrane of the SR, calcium buffering by free proteins (B), and calcium-troponin binding (CaT)

modulated by the muscle-tendon length (X_m) and muscle activation level ($\tilde{A}$) in terms of the constant CaT. To reflect the length dependent changes in calcium sensitivity (31), the forward rate constant (i.e., *K5*) of calcium-troponin reaction was made as a function of X_m to match twitch responses at different X_m as suggested in a previous study (32). The second module (i.e., Module 2) transforms the concentration of CaT relative to the total troponin concentration (T_0) into the level of muscle activation (A). A was determined through exponentiation of $\tilde{A}$ with a single exponent (α) to reflect the degradation of muscle activation for the fluctuation in CaT under physiological conditions. The third module (i.e., Module 3) transforms the muscle activation level (A) resulting from crossbridge formation into the force (F) based on Hill-type mechanics. F was determined via multiplication of the stiffness (K_{SE}) of serial elastic elements, including tendons and aponeuroses, with the variation in X_m reflecting the length- and velocity-tension properties measured under full excitation. The length-tension relationship was proportionally adjusted to the level of muscle activation (A) resulted from neural excitation or electrical stimulation (33). The tension axis of the velocity-tension curve was scaled by the length-tension relationship to reflect the overlap of sliding filaments determining the number of force generating crossbridges (34). The system equations for the canonical model without variation in calcium sensitivity were presented as supplemental information (Supplemental Appendix S1).

[Figure 1 near here]

Dynamics of calcium-force relation

The calcium-force relation was modulated by dynamically varying the two factors that shape the sigmoid steady-state calcium-activation (CaT/T_0-$\tilde{A}_\infty$) relation in module 2 (see Figure 1 and Appendix for the graphical and mathematical description). One factor (*C1*) was the concentration of calcium-binding troponin to reach half of the maximal muscle activation. The other (1/*C2*) was for the slope of the calcium-activation relation at *C1*. *C1* and *C2* were mathematically formulated

as a sigmoidal function that considers the concentration of calcium-binding troponin relative to the total troponin concentration (i.e., CaT/T_0). The equations for the dynamics of *C1* and *C2* are as follows:

$\frac{dCX}{dt} = \frac{CX_{\infty} - CX}{\tau_{CX}}$ where $CX_{\infty} = CXn1 \cdot \left\{1 + tanh\left(\frac{\frac{CaT}{T_0} - CXn2}{CXn3}\right)\right\} + CXi$ and $\tau_{CX} = CXn4$

where *X* indicates 1 for *C1* and 2 for *C2*; *CXn1*, *CXn2*, *CXn3* and *CXi* determine the saturation limit, half maximum, slope and initial value of the CX_{∞} curve, respectively; and *CXn4* is the parameter for the time constant of *CX* dynamics.

Parameter setting

First, the parameters of module 3 were determined from the data for length- and velocity-tension properties under full excitation. K_{SE} for the stiffness of serial elastic elements was estimated by dividing the difference in force (i.e., 30.6 N for CAT14 and 9.5 N for CAT12) by the variation in muscle-tendon length (i.e., 1.9 mm for CAT14 and 1.55 mm for CAT12) and then normalized with the peak isometric force ($P_{0.5}$) at $X_{m,0.5}$ (i.e., 100.3 N for CAT14 and 17.5 N for CAT12). g_1-g_3 for the force-length property were determined by fitting three length-tension datasets (i.e., (0 mm, 62.3 N), (5 mm, 100.3 N), and (10 mm, 115.9 N) for CAT14 and (-0.55 mm, 5.43 N), (3.64 mm, 17.5 N), and (7.9 mm, 30 N) for CAT12) using the function (nlinfit) built in MATLAB software. a_0-d_0 for the velocity-tension property were determined to capture four velocity-tension datasets (i.e., (-74 mm/s, 57.4 N), (-1 mm/s, 99.3 N), (1 mm/s, 101.3 N), and (71 mm/s, 127.4 N) for CAT14 and (-63.81 mm/s, 1.02 N), (-1.5 mm/s, 17.3 N), (1 mm/s, 17.7 N), and (63.81 mm/s, 36.03 N) for CAT12) using the inverse equations derived in a previous study (Eqs. (13)-(16), respectively, in (20)). Second, all parameter values of module 1 except τ_1 and τ_2 for the rate of calcium release from sarcoplasmic reticulum were adopted from the previous study in (20). τ_1 and τ_2 of module 1 and *C1i*, *C2i*, *C3-C5* and α of module 2 were simultaneously optimized to fit the shape of the twitch and tetanic responses during isometric contraction at $X_{m,0.5}$ for the stimulation frequencies of 10 and 40 Hz for CAT14 and 20 and 40

Hz for CAT12, without considering sag behavior by setting *C1n1* and *C2n1* to 0. The force data obtained at 20 Hz for CAT14 and 10 Hz for CAT12 was not involved because the muscle showed the strongest sag behavior under this stimulation frequency. Third, *C1n1-C1n4* in module 2 were first optimized to replicate the sag behavior observed during tetanic response at 20 Hz for CAT14 and 10 Hz for CAT12 and then *C2n1-C2n4* were further optimized for tetanic response at 40 Hz for both CAT14 and CAT12 under isometric conditions at $X_{m,0.5}$. The optimization of parameter values was performed using the optimization tool (i.e., Multiple Run Fitter based on PRAXIS algorithm) built in NEURON software (35). Finally, φ_1- φ_4 of module 1 for the length dependent changes in calcium sensitivity were determined to match the amplitude of the twitch at the physiologically minimal, intermediate and maximal muscle lengths using the polyfit function based on the least squares method built in MATLAB software. All parameter values used for the model of medial gastrocnemius muscle are presented in Table 1.

[Table 1 near here]

Comparison of experimental and simulation data

For the purpose of comparison between different cat MG muscles, all active forces measured experimentally were normalized with $P_{0.5}$ (36). The error between the experimental and simulation data was quantitatively compared across different levels of current stimulation by calculating the root mean square error normalized with maximal force generation using the following equation:

$$\mathrm{NRMSE}\ (\%) = \frac{\sqrt{\frac{1}{N}\sum_{i=1}^{N}(F_m - F_s)^2}}{|max(F_m) - min(F_m)|} \times 100$$

where the subscripts m and s and N indicate measurement, simulation, and the number of samples considered for evaluation, respectively.

Numerical approaches

The model of fast muscle was implemented using the NEURON model description language (NMODL) in the NEURON software environment (version 6.1). The numerical integration of model equations was performed using the integration method (cnexp) built in NEURON with a fixed time step of 0.025 ms. Under this simulation condition, the stability and accuracy of simulations were confirmed while varying parameter values over a wide range. The initial values for Ca and CS in the SR and B and T in the SP were set to 2.5 mM, 30 mM, 0.43 mM, and 70 μM, respectively, based on a previous experimental study (37). The codes for simulations of the muscle-tendon model developed in this study were presented as supplemental information (Supplemental data S1) and will also be available at the public repository of ModelDB (https://senselab.med.yale.edu/modeldb/).

Results

We constructed a computational muscle-tendon model comprising three submodules to demonstrate whether and how dynamical alterations in the calcium-force relation of the myofilaments could explain force variations experimentally observed in fast skeletal muscles under physiological conditions (Figure 1). The calcium sensitivity of muscle force was represented as the steady-state relation of the muscle activation level (i.e., $\tilde{A}_{\infty}$) to the constant concentration of calcium-binding troponin (i.e., CaT/T_0) in the modular model for skeletal muscle (module 2 in Figure 1 A). The CaT/T_0 for half-maximal activation (i.e., $C1$) and the slope at the CaT/T_0 for half-maximal activation (i.e., $1/C2$) in the $\tilde{A}_{\infty}$–CaT/T_0 relation were dynamically varied to infer the dynamic alterations in calcium sensitivity during force production under physiological conditions. The $C1$ and $C2$ variations were modulated as a function of CaT/T_0 considering the saturation (i.e., $C1n1$ and $C2n1$), threshold (i.e., $C1n2$ and $C2n2$), and rate (τ_{C1} and τ_{C2}) in the modular muscle-tendon model (see Figure 1 B for graphical illustration and the Methods for mathematical equations).

Absence of sag behavior under static calcium sensitivity

First, it was evaluated whether or not the model could reproduce the muscle force obtained during isometric contractions at the intermediate length (i.e., $X_{m,0.5}$ = 5 mm) between the physiological maximum (i.e., $X_{m,1}$ = 10 mm) and minimum (i.e., $X_{m,0}$ = 0 mm) over a full physiological range of stimulation frequencies (i.e., 1~40 Hz) with no change in the calcium-force relation (Figure 2). This condition was implemented by setting the value of $C1n1$ and $C2n1$ to zero. The parameters (i.e., τ_1 and τ_2 of module 1 and $C1i$, $C2i$, $C3$, $C4$, $C5$ and α of module 2) shaping the static calcium sensitivity were optimized to match the experimental data showing no sag phenomenon obtained at 1, 10, 40 Hz from the medial gastrocnemius of a cat. The model with the static calcium-force

relation could predict the time course of force profile measured at 1, 10 and 40 as well as 100 Hz, respectively. Yet, the error between the experimental and simulated forces was maximized at a stimulation frequency of 20 Hz showing the absence of sag phenomenon. This result indicates the necessity of a variation in calcium sensitivity for force production during unfused isometric contractions.

[Figure 2 near here]

Depression of calcium sensitivity under low excitation

Then, we assessed if the error between the experiment and simulation at 20 Hz could be compensated by dynamically varying the calcium-force relation. The parameters (i.e., *C1n1*-*C1n4*) underlying dynamic variation of *C1* were optimized for the force data at the stimulation frequency of 20 Hz. The simulation error at 20 Hz could be dramatically reduced by progressively increasing *C1* (Figure 3). The variation in *1/C2* alone through parameter optimization (i.e., *C2n1*-*C2n4*) for the force data at 20 Hz was not sufficient to reproduce the progressive force decline observed during unfused tetanic contractions at 20 Hz (Supplemental Figure S1). This result indicates that calcium sensitivity might decrease mainly due to an increase in the threshold for crossbridge formation under low levels of muscle excitation. The dynamic increase in *C1* did not affect the force production at stimulation frequencies less than 20 Hz. However, the simulation error was significantly increased under higher levels of stimulation frequency (i.e., > 20 Hz). The dynamic increase in *C1* alone could not explain the time course of force production at 40 Hz.

[Figure 3 near here]

Potentiation of calcium sensitivity under high excitation

Lastly, it was assessed if the increased error between the experiment and simulation with only variation in *C1* for stimulation frequencies > 20 Hz could be compensated by dynamically varying the calcium-force relation. The parameters

(i.e., *C2n1*-*C2n4*) underlying dynamic variation of *1/C2* were further optimized for the force data at the stimulation frequency of 40 Hz. The simulation error with only variation in *C1* for stimulation frequencies > 20 Hz including 100 Hz could be substantially reduced by dynamically increasing the *1/C2* value (Figure 4). The variation in *C1* alone through parameter optimization (i.e., *C1n1*-*C1n4*) for the force data at 40 Hz was not sufficient to reproduce the slow rate of force development following the fast rate of initial force development at 40 Hz (Supplemental Figure S2). This result indicates that the slow force development observed at 40 Hz might be induced by the gradual enhancement of calcium sensitivity mainly through a slow increase in the cooperativity of crossbridge formation under high levels of unfused muscle excitation. In addition, the dynamic steepening of the calcium-force relation did not affect the reproduction of forces at lower stimulation frequencies < 40 Hz. All these results indicate that calcium sensitivity operationally varies depending upon the state of muscle excitation under physiological conditions.

[Figure 4 near here]

Dynamics of calcium sensitivity at the intermediate length

To visualize how the calcium-force relation varies depending on the level of excitation, the steady-state calcium-activation (i.e., CaT/T_0–$\tilde{A}_\infty$) curve was reconstructed at the initial and maximally varied states during isometric contractions at the intermediate muscle-tendon length over the full physiological range of stimulation frequencies (Figure 5 A). The shift of the midpoint of the calcium-activation relation to the right was facilitated as the excitation level increased from 20 Hz. However, the slope of the calcium-activation relation was steepened at higher levels of excitation frequencies from 40 Hz. These findings obtained under steady-state conditions were also confirmed in the transient state for the calcium binding troponin-muscle activation (i.e., CaT/T_0–A) (Figure 5 B) and intracellular calcium-muscle force (i.e., *Ca*–*F*) (Figure 5 C) relation. The

steady-state calcium-activation relations could be reflected in the transient calcium-force relations measured during the relaxation of force production (see the insets in Figure 5 C). These results imply that the increase in the threshold for crossbridge formation can be counteracted by an enhancement in the cooperativity for crossbridge formation as the level of muscle excitation increases.

[Figure 5 near here]

Role of cooperativity in shaping sag behavior

Comparing to the experimental data obtained from the cat medial gastrocnemius, we have shown the capability of the muscle-tendon model for producing a typical form of sag behavior showing progressive force decline after the initial peak during the unfused isometric contraction. It was further assessed whether the model could represent more complex forms of sag behavior that have been reported in previous experimental studies (38, 39). Figure 6 shows the four representative types of sag behavior that are identifiable by the model during unfused isometric contractions at the intermediate length for stimulation frequencies ranging from 20 to 30 Hz. The simple form (referred to as Type I) of sag behavior with progressive force decline after the initial peak could be produced by changing *C1* (Figure 6 A). However, complex forms of sag behavior could be induced by varying *C2* parameters at the same time. With lower threshold (less *C2n2*) and slower speed (larger τ_{C2}) (i.e., 100 ms) in the slope variation of the calcium-force relation, the simple sag behavior transitioned to a complex form (referred to as Type II) of sag behavior, showing multiple force peaks following the initial force peak at 20 Hz (Figure 4 B). With the same *C2n2* but much slower τ_{C2} (i.e., 500 ms) in comparison to the Type II sag, another complex form (referred to as Type III) of sag behavior showing double force declines following the initial force peak was induced at 20 Hz (Figure 6 C). With the same value of *C2n2* and τ_{C2} used for the Type III sag, the last complex form

(referred to as Type IV) of force decline followed by force amplification after the initial force peak was determined to be under 30 Hz (Figure 6 D). It should be noted that the Type I, II and IV sag have been experimentally identified while the Type III sag was newly found through the analysis of the muscle-tendon model in this study.

[Figure 6 near here]

We further investigated how *C2n2*, τ_{C2} and excitation level might interplay to shape the sag behavior during submaximal contractions at the intermediate muscle-tendon length. Figure 7 shows the map of sag types over the parameter space for *C2n2* and τ_{C2} under two different levels (i.e., 20 and 30 Hz) of muscle excitation. Type I sag tended to transition to Type II sag as *C2n2* and τ_{C2} decreased while Type I sag tended to transition to Type III sag as *C2n2* decreased and τ_{C2} increased under the stimulation frequency of 20 Hz. Interestingly, the interaction of *C2n2* and τ_{C2} on sag type differed under the stimulation frequency of 30 Hz. Sag phenomenon tended to disappear as *C2n2* and τ_{C2} decreased while Type I sag tended to transition to Type IV as *C2n2* decreased and τ_{C2} increased. These results indicate that the type of sag phenomenon might be determined predominantly by a dynamic change in the slope of the calcium-force relation depending on the level of excitation.

[Figure 7 near here]

Length and velocity dependence of calcium sensitivity

The dynamic variations in calcium-force relation were further estimated for the force production under full excitation (i.e., 100 Hz) while varying the muscle-tendon length at a constant velocity. The muscle-tendon model reflecting the length dependence of twitch response (i.e., K5 in Module 1 of Figure 1) could accurately demonstrate the characteristics of force-length and force-velocity

behaviors, as seen in the experiment under full excitation (Figure 8). Interestingly, the depression effect of the rightward drift (i.e., *C1*) of the calcium-force relation was sufficiently compensated by the potentiation effect of the upward drift (i.e., *C2*) of the slope in the calcium-force relation under full excitation. Resultantly, the dynamic variation in the calcium-force relation did not tend to affect the force production because the activation level was almost maximal under full excitation. This result suggests that the net effect of both changes in the midpoint and the slope of the calcium-force relation may be crucial for the force-length and force-velocity properties under the full excitation level.

[Figure 8 near here]

Validation of model predictions

It was tested if the results hold for cat MG muscles showing different sag behavior during unfused isometric contractions. The same modeling approach used for CAT14 was applied to the force data independently measured from the different cat MG muscle (i.e., CAT12) with the sag behavior occurring at the lower level of stimulation frequency (i.e., 10 Hz) than CAT14 (see Table 1 for the parameter values of model CAT12). As expected from the model of CAT14, the muscle-tendon model for the new force data could not exhibit the sag behavior without variations in the calcium-force relation during isometric contractions at the intermediate muscle-tendon length (i.e., $X_{m,0.5}$). The parameters for *C1* dynamics (i.e., *C1n1*-*C1n4*) and *C2* dynamics (i.e., *C2n1*-*C2n4*) were optimized for the new force data at the stimulation frequency of 10 and 40 Hz, respectively. The midpoint (i.e., *C1*) of the calcium-force relation was required to drift rightward whereas the slope (i.e., *1/C2*) of the calcium-force relation needed to drift upward in order to reproduce the isometric forces over a full range of stimulation frequencies (i.e., 1 – 100 Hz) at $X_{m,0.5}$, including sag behavior (Supplemental Figure S3). The pattern of dynamic alternations in the calcium-force relation was

similar to that inferred from the data set for CAT14 (Supplemental Figure S4). All types of sag behavior identified by the model CAT14 were also found by varying the parameters (i.e., *C2n1* and *C2n2*) for the slope variation of the calcium-force relation in the model of CAT12 (Supplemental Figure S5). In addition, the model CAT12 reflecting the length dependent twitch response could also replicate the force-length and force-velocity properties under full excitation showing the importance of net effect of *C1* and *C2* variation in the calcium-force relation (Supplemental Figure S6). With this end, the characteristics of dynamic alternations in the calcium-force relation seem to be similar across fast-type muscles with sag behavior in adult cats.

Discussion

Calcium sensitivity under physiological conditions

The dynamics of calcium sensitivity during force production differed depending upon the muscle type (slow-twitch versus fast-twitch). In contrast to the case for slow muscles such as soleus (20), our model of the cat medial gastrocnemius muscle revealed that the calcium sensitivity should dynamically change during force generation over a physiological range of stimulation frequencies (20~40 Hz) and muscle-tendon lengths (0~10 mm) in fast skeletal muscle. The progressive shift (i.e., *C1*) of the current-force relation to the right was required to replicate the time course of force decline measured at a stimulation frequency of 20 Hz (Figure 3). In addition, to reproduce the force production under stimulation frequencies over 40 Hz, the slope (i.e., *1/C2)* of the calcium-force relation must be steepened to counteract the decrease in muscle activation by the rightward shift of the calcium-force relations over time (Figure 4). Furthermore, the slope variation in the calcium-force relation played a crucial role in determining the form of sag behavior (Figures 6 and 7).

The static calcium-force relation has been analyzed to infer the calcium sensitivity of force producing crossbridge formation under in vitro steady-state conditions (40). Our study may provide insights into the dynamic aspects regarding the calcium sensitivity of crossbridge formation under physiological conditions (Figure 5). That is, the calcium sensitivity of crossbridge formation might tend to be constant under low levels of muscle excitation (i.e., < 10 Hz). However, the calcium sensitivity of crossbridge formation might be progressively lowered, resulting in sag behavior under intermediate levels of muscle excitation (i.e., approximately 20 Hz), and might be progressively increased, resulting in force enhancement under high levels of muscle excitation (i.e., > 40 Hz). All these findings suggest that the calcium sensitivity of crossbridge formation might operationally vary depending on both neural excitation and muscle length in fast muscle under physiological conditions.

Comparison with previous studies

Slow- and fast-twitch muscle fibers have been shown to correspond to Type I and Type II chemically identified by myosin ATPase or MHC I and MHC II by myosin heavy chain isoform (41). The type-specific compositions of myosin proteins affecting crossbridge cycling have been suggested to underlie slow but sustained contractions of slow muscle fibers and fast but fatiguing contractions of fast muscle fibers (42, 43). These findings from isolated muscle fibers might also account for the differential (fast but activity dependent) dynamics of calcium sensitivity in fast muscles compared to slow muscles that we demonstrated during force production under physiological conditions in this study. Further studies are warranted to confirm the correlation between type-specific myosin proteins and calcium sensitivity dynamics in isolated muscle fibers under physiologically relevant calcium excitations.

Previous experimental studies have shown a rightward midpoint shift and a decreased slope steepness in the calcium-force relation as the inorganic phosphate (P_i) concentration increases in isolated muscle fibers (18). As shown in the previous study, the midpoint (i.e., *C1*) of the calcium-force relation was required to shift to the right as the excitation level increased in the present study. In contrast to the previous study, however, the slope (i.e., *1/C2*) of the calcium-force relation needed to be steepened as the excitation level increased under physiological conditions (Figure 4). This result suggests that the cooperativity of crossbridge formation might be enhanced through other factors, such as strain-induced potentiation (44, 45), counteracting the inhibitory effects of P_i accumulation at high excitation levels in fast muscle under physiological conditions.

Sag behavior has been investigated in fast muscles in cats (21), rats (38), frogs (46) and humans (47). A variety of sag forms have been identified from rat triceps surae motor units, including one simple form and two complex forms of

the sag phenomenon (38, 39). In this study, our model could reproduce all three forms of the sag phenomenon, suggesting that the simple form (i.e., Type I) tended to be determined by a rightward shift (i.e., *C1*) of the calcium-force relation, whereas the complex forms (i.e., Type II & Iv) tended to be shaped by the slope variation (i.e., *1/C2*) in the calcium-force relation (Figure 6). The muscle-tendon model could further identify different types (e.g., Type III) of sag behavior while varying the excitation level and the parameters (i.e., *C2n2* and τ_{C2}) for the threshold and speed in the slope variation of the calcium-force relation (Figure 7). This result suggests that the interplay between the excitation level and the slope variation in the calcium-force relation plays a critical role in determining the form of the sag phenomenon.

In intact animal preparations, the sag magnitude has been shown to tend to be greater at shortened lengths than at lengthened lengths (48). In isolated muscle fiber preparations, the slope of the steady-state calcium-force relation has been reported to decrease as the muscle length is stretched (7). Thus, we tested whether the length dependence of sag magnitude could be explained by the slope variation of calcium-force relation during isometric contractions at 20 Hz for CAT14 and 10 Hz for CAT12. The model without slope variation in the calcium-force relation exhibited the increasing sag amplitude with increasing muscle length during isomeric contractions at 20 Hz (Figure 9 A1 and B1 for CAT14 and Supplemental Figure S7 A1 and B1 for CAT12). When the slope of the calcium-force relation was varied by lowering *C2n2* and decreasing *C2n1* at the shortened state and increasing *C2n1* at the lengthened state from the default value for the intermediate state as reported from skinned fiber experiments (7), however, the model muscle displayed the opposite tendency showing the decreasing sag amplitude with increasing muscle length (Figure 9 A2 and B2 for CAT14 and Supplemental Figure S7 A2 and B2 for CAT12). Resultantly, the length dependent sag magnitude could be explained by the progressively lowered calcium sensitivity at shortened muscle lengths but progressively increased calcium sensitivity at lengthened muscle lengths under intermediate levels of muscle excitation. This result highlighted the interplay between the

slope of calcium-force relation and muscle length in shaping the sag behavior under physiological conditions.

[Figure 9 near here]

The computational model for fast skeletal muscle has been developed phenomenologically, focusing on capturing force-length and force-velocity properties across different levels of muscle excitation (48) or superposition of twitch responses under isometric conditions (49, 50). More recently, it has been proposed that the cooperativity of crossbridge formation should be incorporated into the phenomenological model to reflect the physiological conditions of muscle activation dynamics (51). In the present study, we have further demonstrated that the dynamic variation in the calcium-force relation must be considered to reproduce the complex forms of force production experimentally shown in fast muscle studies under submaximal rather than maximal contractions (see Figure 8 for the force-length and force-velocity properties under full excitation). Therefore, the muscle-tendon model developed for this study may provide a physiologically plausible framework for force production over a full physiological range of stimulation frequencies and muscle lengths under isometric and isokinetic conditions.

Current limitations

The model including the dynamic variation in calcium-force relationship could reproduce force production of a cat medial gastrocnemius muscle over a full physiological range of stimulation frequencies and muscle lengths under isometric and isokinetic conditions. However, this model should be further improved to reflect multiple factors determining the physiological behaviors of fast muscles. First, in the present study, the mechanisms controlling intracellular calcium concentration (Module 1) were assumed not to change during

submaximal muscle contractions over short periods of time (i.e., < 1 second). However, this assumption may not hold for maximal muscle contractions over longer periods of time (i.e., > several seconds) since under this condition the intracellular calcium concentration has been reported to increase and then decrease during progressive force decline or fatigue (23). Second, the calcium-force relationship was dynamically varied by directly manipulating the parameters of the curve (Module 2). However, the modular modeling approach used for this study could be extended to systematically evaluate potential subcellular mechanisms that might underly the dynamic variation in calcium sensitivity during force production in fast muscles (52). Third, the calcium dynamics in the sarcoplasm were modeled based on the experimental data obtained from rats in the present model. Further experimental investigations are needed to identify the specific differences in sarcoplasmic calcium dynamics between different species and muscle types (53). Fourth, the modeling approach used for this study represents the average behavior of whole muscle comprising different muscle fibers. Although cat gastrocnemius muscles are known to be comprised predominantly of fast-twitch muscle fibers (i.e., over 75 %) (30), this limitation might increase the error between the simulation and experimental data. Future studies should be performed to see how the heterogeneous organization of whole muscle influences the force output (19). Fifth, the current model was validated only for constant stimulation frequencies. Neural excitation has been shown to be random rather than constant (54). Thus, the current model might not reflect the random effect of neural excitation on force production, such as the catch-like property (55). Last, the present model is limited to force production under isometric and isokinetic muscle conditions. Future experiments are needed to measure the data on force production during dynamic variations in muscle length, such as locomotor-like movements (28).

Conclusions

Unlike in slow-twitch muscles, the calcium-force relationship needed to dynamically change to replicate the force responses of fast-twitch muscles over a full physiological range of stimulation frequencies and muscle lengths. The dynamic variation in the calcium-force relation operationally varied depending upon the excitation level and muscle length during unfused isometric contractions, whereas it did not tend to affect the length- and velocity-tension properties under full excitation. The slope variation in the calcium-force relationship was crucial to determine the types and the length dependence of sag phenomena. These results provide insights into the dynamic properties of crossbridge formation during force production and a computational framework for physiologically realistic modeling of skeletal muscles.

Author Contributions

HK and CJ conceived and designed the study; HK performed the simulations; HK and CJ analyzed the data; HK prepared the draft of manuscript; HK and CJ edited and approved the manuscript.

Competing Interest Statement

No potential competing interest was reported by the authors.

Acknowledgments

The authors truly thank Dr. Thomas Sandercock for performing the experiment and data collection from cat medial gastrocnemius muscles and valuable comments on the manuscript. This study was supported by the National Institutes of Health Grant R01 (NS071951 and NS62200) and the DGIST R&D Program (21-BT-06) and National Research Foundation of Korea (2021060027) funded by the Ministry of Science and ICT.

References

1. Spudich JA. The myosin swinging cross-bridge model. Nat Rev Mol Cell Biol. 2001;2(5):387-92.

2. Gollapudi SK, Lin DC. Experimental determination of sarcomere force-length relationship in type-I human skeletal muscle fibers. J Biomech. 2009;42(13):2011-6.

3. Winters TM, Takahashi M, Lieber RL, Ward SR. Whole muscle length-tension relationships are accurately modeled as scaled sarcomeres in rabbit hindlimb muscles. J Biomech. 2011;44(1):109-15.

4. Bershitsky SY, Tsaturyan AK, Bershitskaya ON, Mashanov GI, Brown P, Burns R, et al. Muscle force is generated by myosin heads stereospecifically attached to actin. Nature. 1997;388(6638):186-90.

5. Lehman W, Craig R, Vibert P. Ca(2+)-induced tropomyosin movement in Limulus thin filaments revealed by three-dimensional reconstruction. Nature. 1994;368(6466):65-7.

6. Ridgway EB, Gordon AM, Martyn DA. Hysteresis in the force-calcium relation in muscle. Science. 1983;219(4588):1075-7.

7. Endo M. Stretch-induced increase in activation of skinned muscle fibres by calcium. Nat New Biol. 1972;237(76):211-3.

8. Brandt PW, Cox RN, Kawai M. Can the binding of Ca2+ to two regulatory sites on troponin C determine the steep pCa/tension relationship of skeletal muscle? Proc Natl Acad Sci U S A. 1980;77(8):4717-20.

9. Julian FJ. The effect of calcium on the force-velocity relation of briefly glycerinated frog muscle fibres. J Physiol. 1971;218(1):117-45.

10. Gordon AM, Homsher E, Regnier M. Regulation of contraction in striated muscle. Physiol Rev. 2000;80(2):853-924.

11. Scott W, Stevens J, Binder-Macleod SA. Human skeletal muscle fiber type classifications. Phys Ther. 2001;81(11):1810-6.

12. Donaldson SK. Ca2+-activated force-generating properties of mammalian skeletal muscle fibres: histochemically identified single peeled rabbit fibres. J Muscle Res Cell Motil. 1984;5(6):593-612.

13. Fink RH, Stephenson DG, Williams DA. Calcium and strontium activation of single skinned muscle fibres of normal and dystrophic mice. J Physiol. 1986;373:513-25.
14. Lucas SM, Ruff RL, Binder MD. Specific tension measurements in single soleus and medial gastrocnemius muscle fibers of the cat. Exp Neurol. 1987;95(1):142-54.
15. Takagi A, Endo M. Guinea pig soleus and extensor digitorum longus: a study on single-skimmed fibers. Exp Neurol. 1977;55(1):95-101.
16. Danieli-Betto D, Betto R, Midrio M. Calcium sensitivity and myofibrillar protein isoforms of rat skinned skeletal muscle fibres. Pflugers Arch. 1990;417(3):303-8.
17. Ruff RL. Calcium sensitivity of fast- and slow-twitch human muscle fibers. Muscle Nerve. 1989;12(1):32-7.
18. Debold EP, Romatowski J, Fitts RH. The depressive effect of Pi on the force-pCa relationship in skinned single muscle fibers is temperature dependent. Am J Physiol Cell Physiol. 2006;290(4):C1041-50.
19. Burke RE. Motor Units - Physiological-Histochemical Profiles, Neural Connectivity and Functional Specializations. American Zoologist. 1978;18(1):127-34.
20. Kim H, Sandercock TG, Heckman CJ. An action potential-driven model of soleus muscle activation dynamics for locomotor-like movements. J Neural Eng. 2015;12(4):046025.
21. Burke RE, Levine DN, Tsairis P, Zajac FE, 3rd. Physiological types and histochemical profiles in motor units of the cat gastrocnemius. J Physiol. 1973;234(3):723-48.
22. Smith IC, Bellissimo C, Herzog W, Tupling AR. Can inorganic phosphate explain sag during unfused tetanic contractions of skeletal muscle? Physiol Rep. 2016;4(22).
23. Allen DG, Lamb GD, Westerblad H. Impaired calcium release during fatigue. J Appl Physiol (1985). 2008;104(1):296-305.

24. Tupling AR. The decay phase of Ca2+ transients in skeletal muscle: regulation and physiology. Appl Physiol Nutr Metab. 2009;34(3):373-6.
25. Allen DG, Lamb GD, Westerblad H. Skeletal muscle fatigue: cellular mechanisms. Physiol Rev. 2008;88(1):287-332.
26. Sandercock TG, Heckman CJ. Force from cat soleus muscle during imposed locomotor-like movements: experimental data versus Hill-type model predictions. J Neurophysiol. 1997;77(3):1538-52.
27. Goslow GE, Jr., Reinking RM, Stuart DG. The cat step cycle: hind limb joint angles and muscle lengths during unrestrained locomotion. J Morphol. 1973;141(1):1-41.
28. Goslow GE, Jr., Reinking RM, Stuart DG. Physiological extent, range and rate of muscle stretch for soleus, medial gastrocnemius and tibialis anterior in the cat. Pflugers Arch. 1973;341(1):77-86.
29. Herzog W, Leonard TR, Renaud JM, Wallace J, Chaki G, Bornemisza S. Force-length properties and functional demands of cat gastrocnemius, soleus and plantaris muscles. J Biomech. 1992;25(11):1329-35.
30. Burke RE, Tsairis P. Anatomy and innervation ratios in motor units of cat gastrocnemius. J Physiol. 1973;234(3):749-65.
31. Martyn DA, Coby R, Huntsman LL, Gordon AM. Force-calcium relations in skinned twitch and slow-tonic frog muscle fibres have similar sarcomere length dependencies. J Muscle Res Cell Motil. 1993;14(1):65-75.
32. Williams T, Bowtell G, Curtin NA. Predicting force generation by lamprey muscle during applied sinusoidal movement using a simple dynamic model. J Exp Biol. 1998;201 (Pt 6):869-75.
33. Rack PM, Westbury DR. The effects of length and stimulus rate on tension in the isometric cat soleus muscle. J Physiol. 1969;204(2):443-60.
34. Gordon AM, Huxley AF, Julian FJ. The variation in isometric tension with sarcomere length in vertebrate muscle fibres. J Physiol. 1966;184(1):170-92.
35. Hines ML, Carnevale NT. The NEURON simulation environment. Neural Comput. 1997;9(6):1179-209.

36. Zajac FE. Muscle and tendon: properties, models, scaling, and application to biomechanics and motor control. Crit Rev Biomed Eng. 1989;17(4):359-411.

37. Westerblad H, Allen DG. The role of sarcoplasmic reticulum in relaxation of mouse muscle; effects of 2,5-di(tert-butyl)-1,4-benzohydroquinone. J Physiol. 1994;474(2):291-301.

38. Carp JS, Herchenroder PA, Chen XY, Wolpaw JR. Sag during unfused tetanic contractions in rat triceps surae motor units. J Neurophysiol. 1999;81(6):2647-61.

39. Krysciak K, Celichowski J, Krutki P, Raikova R, Drzymala-Celichowska H. Factors contributing to sag in unfused tetanic contractions of fast motor units in rat medial gastrocnemius. J Electromyogr Kinesiol. 2019;44:70-7.

40. Wahr PA, Metzger JM. Role of Ca2+ and cross-bridges in skeletal muscle thin filament activation probed with Ca2+ sensitizers. Biophys J. 1999;76(4):2166-76.

41. Schiaffino S, Reggiani C. Fiber types in mammalian skeletal muscles. Physiol Rev. 2011;91(4):1447-531.

42. Metzger JM, Moss RL. Calcium-sensitive cross-bridge transitions in mammalian fast and slow skeletal muscle fibers. Science. 1990;247(4946):1088-90.

43. Stehle R, Kraft T. Cross-bridges in slow skeletal muscle: not only slow but also soft. J Physiol. 2018;596(7):1121-2.

44. Frigon A, Thompson CK, Johnson MD, Manuel M, Hornby TG, Heckman CJ. Extra forces evoked during electrical stimulation of the muscle or its nerve are generated and modulated by a length-dependent intrinsic property of muscle in humans and cats. J Neurosci. 2011;31(15):5579-88.

45. MacIntosh BR. Cellular and whole muscle studies of activity dependent potentiation. Adv Exp Med Biol. 2010;682:315-42.

46. Allen DG, Lannergren J, Westerblad H. Muscle cell function during prolonged activity: cellular mechanisms of fatigue. Exp Physiol. 1995;80(4):497-527.

47. Smith IC, Ali J, Power GA, Herzog W. The sag response in human muscle contraction. Eur J Appl Physiol. 2018;118(5):1063-77.

48. Brown IE, Loeb GE. Measured and modeled properties of mammalian skeletal muscle: IV. dynamics of activation and deactivation. J Muscle Res Cell Motil. 2000;21(1):33-47.

49. Fuglevand AJ, Winter DA, Patla AE. Models of recruitment and rate coding organization in motor-unit pools. J Neurophysiol. 1993;70(6):2470-88.

50. Petersen E, Rostalski P. A Comprehensive Mathematical Model of Motor Unit Pool Organization, Surface Electromyography, and Force Generation. Front Physiol. 2019;10:176.

51. Nagamori A, Laine CM, Loeb GE, Valero-Cuevas FJ. Force variability is mostly not motor noise: Theoretical implications for motor control. PLoS Comput Biol. 2021;17(3):e1008707.

52. MacIntosh BR. Role of calcium sensitivity modulation in skeletal muscle performance. News in Physiological Sciences. 2003;18:222-5.

53. Berchtold MW, Brinkmeier H, Muntener M. Calcium ion in skeletal muscle: its crucial role for muscle function, plasticity, and disease. Physiol Rev. 2000;80(3):1215-65.

54. Moritz CT, Barry BK, Pascoe MA, Enoka RM. Discharge rate variability influences the variation in force fluctuations across the working range of a hand muscle. J Neurophysiol. 2005;93(5):2449-59.

55. Binder-Macleod S, Kesar T. Catchlike property of skeletal muscle: recent findings and clinical implications. Muscle Nerve. 2005;31(6):681-93.

Figure legends

Figure 1 Schematic diagram of the action potential-driven muscle-tendon model. **A.** Muscle activation dynamics (*A*) in response to action potentials (*ESs*) from spinal motoneurons (dotted line box) and muscle mechanics (*F*) induced by contractile elements (*CEs*) and serial elastic elements (*SEs*). X_{CE} and X_m show the length of the contractile element and entire muscle-tendon unit. *C1* and *1/C2* indicate the constant concentration of calcium-binding troponin relative to the total troponin concentration (CaT/T_0) for the half-maximal level of steady-state muscle activation ($\tilde{A}_\infty$) and the slope of the CaT/T_0–$\tilde{A}_\infty$ curve at *C1*. **B.** Relationship of steady-state *C1* ($C1_\infty$) and *C2* ($C2_\infty$) with the concentration of CaT/T_0 (left panel) and time constants (τ_{C1} and τ_{C2}) for the *C1* and *C2* dynamics (right panel). *C1n1* and *C2n1* limit the saturation level, and *C1n2* and *C2n2* indicate a constant concentration of CaT/T_0 for the midpoint between the minimum and maximum in the $C1_\infty$ and $C2_\infty$ curves.

Figure 2 Tetani of the muscle-tendon model without changes in *C1* and *C2* in response to various input frequencies under isometric contraction at $X_{m,0.5}$ for CAT14. **A.** Twitch (upper) and current stimulation (bottom). **B.** Unfused tetanus (upper) and current stimulation (10 Hz, bottom). **C.** Unfused tetanus (upper) and current stimulation (20 Hz, bottom). **D.** Unfused tetanus (upper) and current stimulation (40 Hz, bottom). **E.** Fused tetanus (upper) and current stimulation (100 Hz, bottom). **F.** Overall error value between simulated and experimental force data for the panels A-E. Black and blue lines indicate the data obtained from the experiment and simulation.

Figure 3 Tetani of the muscle-tendon model with changes only in *C1* in response to various input frequencies under isometric contraction at $X_{m,0.5}$ for CAT14. **A.** Twitch (upper), change in *C1* (middle) and current stimulation (bottom). **B.** Unfused tetanus (upper), change in *C1* (middle) and current stimulation (10 Hz, bottom). **C.** Unfused tetanus (upper), change in *C1* (middle) and current stimulation (20 Hz, bottom). **D.** Unfused tetanus (upper), change in *C1* (middle) and current stimulation (40 Hz, bottom). **E.** Fused tetanus (upper), change in *C1*

(middle) and current stimulation (100 Hz, bottom). **F.** Overall error value between simulated and experimental force data for the panels **A**-**E**. Black and blue lines indicate the data obtained from the experiment and simulation.

Figure 4 Tetani of the muscle-tendon model with changes in both *C1* and *C2* under isometric contraction at $X_{m,0.5}$ for CAT14. **A.** Twitch (upper), change in *C1* & *C2* (middle) and current stimulation (bottom). **B.** Unfused tetanus (upper), change in *C1* & *C2* (middle) and current stimulation (10 Hz, bottom). **C.** Unfused tetanus (upper), change in *C1* & *C2* (middle) and current stimulation (20 Hz, bottom). **D.** Unfused tetanus (upper), change in *C1* & *C2* (middle) and current stimulation (40 Hz, bottom). **E.** Fused tetanus (upper), change in *C1* & *C2* (middle) and current stimulation (100 Hz, bottom). **F.** Simulation error with no variation in *C1* & *C2* (black), only variation in *C1* (gray) and variation in both *C1* & *C2* (white) at the stimulation frequency of 1 Hz (A), 10 Hz (B), 20 Hz (C), 40 Hz (D) and 100 Hz (E). Black and blue lines in **A**-**E** indicate the data obtained from the experiment and simulation with variation in both *C1* & *C2*. The comparison of experimental and simulated data for the case of no variation in *C1* & *C2* and only variation in *C1* was presented in Figure 2 and 3.

Figure 5 Variation in the calcium-force relation during isometric force production at $X_{m,0.5}$ for CAT14. **A**. Steady-state relation of muscle activation ($\tilde{A}_{\infty}$) to calcium binding troponin relative to the total troponin concentration (CaT/T_0) in the initial and maximally varied states at various stimulation frequencies. **B.** Transient relation of muscle activation (A) to CaT/T_0 at various levels of stimulation frequency. **C.** Transient relation of muscle force (F) and sarcoplasmic calcium (Ca) at various levels of stimulation frequency. Insets indicate the transient relationship between calcium and activation (**B**) and calcium and force (**C**) on the relaxation phase of force production.

Figure 6 Representative types of sag behavior at $X_{m,0.5}$ for CAT14. **A.** Simple sag form at 20 Hz current stimulation (left) and changes in *C1* and *C2* (right) with default values of *C2n2* and τ_{C2}. **B-D.** Complex sag forms at 20 Hz (**B** and **C**) and 30 Hz (**D**) stimulation frequencies (left) and changes in *C1* and *C2* (right) with

variations in *C2n2* and τ_{C2}. Arrows indicate the direction of force production after the initial peak force.

Figure 7 Type map of sag behavior with changes in threshold (*C2n2*) and time constant (τ_{C2}) for the slope (*C2*) variation in the calcium-activation ($CaT/T_0 - \tilde{A}_{\infty}$) relation at $X_{m,0.5}$ for CAT14. **A.** Type map under 20 Hz current stimulation. **B.** Type map under 30 Hz current stimulation.

Figure 8 Length- and velocity-force properties of the model CAT14 with changes in *C1* and *C2*. **A.** Force responses during lengthening (upper), changes in *C1* and *C2* (middle) and current stimulation (bottom). **B.** Force responses during shortening (upper), changes in *C1* and *C2* (middle) and current stimulation (bottom). **C.** Force responses during step lengthening (upper), changes in *C1* and *C2* (middle) and current stimulation (bottom). **D.** Profiles of the muscle-tendon length variation for **A**, **B** and **C**. Black and blue lines indicate the experimental and simulated data.

Figure 9 Length dependence of the sag magnitude in the model of CAT14. **A1 & B1.** Unfused tetanus (upper), changes in *C1* and *C2* (middle) and 20 Hz current impulse stimulation (bottom) with no change in the slope of calcium-force relation at the physiologically minimal (0 mm) and maximal (10 mm) muscle-tendon length. **A2 & B2.** Unfused tetanus (upper), changes in *C1* and *C2* (middle) and 20 Hz current impulse stimulation (bottom) with slope variation in the calcium-force relation at the physiologically minimal and maximal muscle-tendon length. Black and blue lines indicate the experimental and simulated data. Arrows indicate the degree of force decline after the initial peak force.

Figure S1 Tetani of the model CAT14 with changes only in *C2* during isometric contraction at the $X_{m,0.5}$ (i.e., 5 mm). Unfused tetanus (upper), change in *C2* (middle) and current stimulation (20 Hz, bottom). Black and blue lines indicate the data obtained from the experiment and simulation.

Figure S2 Tetani of the model CAT14 with changes only in *C1* during isometric contraction at the $X_{m,0.5}$ (i.e., 5 mm). Unfused tetanus (upper), change in *C1*

(middle) and current stimulation (40 Hz, bottom). Black and blue lines indicate the data obtained from the experiment and simulation.

Figure S3 Tetani of the model CAT12 with changes in both *C1* and *C2* under isometric contraction at $X_{m,0.5}$. **A.** Twitch (upper), change in *C1* & *C2* (middle) and current stimulation (bottom). **B.** Unfused tetanus (upper), change in *C1* & *C2* (middle) and current stimulation (10 Hz, bottom). **C.** Unfused tetanus (upper), change in *C1* & *C2* (middle) and current stimulation (20 Hz, bottom). **D.** Unfused tetanus (upper), change in *C1* & *C2* (middle) and current stimulation (40 Hz, bottom). **E.** Fused tetanus (upper), change in *C1* & *C2* (middle) and current stimulation (100 Hz, bottom). **F.** Simulation error with no variation in *C1* & *C2* (black), only variation in *C1* (gray) and variation in both *C1* & *C2* (white) at the stimulation frequency of 1 Hz (A), 10 Hz (B), 20 Hz (C), 40 Hz (D) and 100 Hz (E). Black and blue lines in **A**-**E** indicate the data obtained from the experiment and simulation with variation in both *C1* & *C2*.

Figure S4 Variation in the calcium-force relation during isometric force production at $X_{m,0.5}$ for CAT12. **A**. Steady-state relation of muscle activation ($\tilde{A}_{\infty}$) to calcium binding troponin relative to the total troponin concentration (CaT/T_0) in the initial and maximally varied states at various stimulation frequencies. **B.** Transient relation of muscle activation (*A*) to CaT/T_0 at various levels of stimulation frequency. **C.** Transient relation of muscle force (*F*) and sarcoplasmic calcium (*Ca*) at various levels of stimulation frequency. Insets indicate the transient relationship between calcium and activation (**B**) and calcium and force (**C**) on the relaxation phase of force production.

Figure S5 Representative types of sag at $X_{m,0.5}$ for CAT12. **A.** Simple sag form at 20 Hz current stimulation (left) and changes in *C1* and *C2* (right) with default values of *C2n2* and τ_{C2}. **B-D.** Complex sag forms at 20 Hz (**B** and **C**) and 30 Hz (**D**) stimulation frequencies (left) and changes in *C1* and *C2* (right) with variations in *C2n2* and τ_{C2}. Arrows indicate the direction of force production after the initial peak force.

Figure S6 Length- and velocity-force properties of the model CAT12 with changes in *C1* and *C2*. **A.** Force responses during lengthening (upper), changes in *C1* and *C2* (middle) and current stimulation (bottom). **B.** Force responses during shortening (upper), changes in *C1* and *C2* (middle) and current stimulation (bottom). **C.** Force responses during step lengthening (upper), changes in *C1* and *C2* (middle) and current stimulation (bottom). **D.** Profiles of the muscle-tendon length variation for **A**, **B** and **C**. Black and blue lines indicate the experimental and simulated data.

Figure S7 Length dependence of the sag magnitude in the model of CAT12. **A1 & B1.** Unfused tetanus (upper), changes in *C1* and *C2* (middle) and 20 Hz current impulse stimulation (bottom) with no change in the slope of calcium-force relation at the physiologically minimal (-0.55 mm) and maximal (7.9 mm) muscle-tendon length. **A2 & B2.** Unfused tetanus (upper), changes in *C1* and *C2* (middle) and 20 Hz current impulse stimulation (bottom) with slope variation in the calcium-force relation at the physiologically minimal and maximal muscle-tendon length. Black and blue lines indicate the experimental and simulated data. Arrows indicate the degree of force decline after the initial peak force.

Table 1 Parameter values of fast muscle model for cat medial gastrocnemius muscles (CAT14 for model development and CAT12 for model validation).

Module	Parameter	CAT14	CAT12
		Value	Value
Module 1	K1 [$M^{-1}\cdot ms^{-1}$]	3000	3000
	K2 [ms^{-1}]	3	3
	K3 [$M^{-1}\cdot ms^{-1}$]	400	400
	K4 [ms^{-1}]	1	1
	K5i [$M^{-1}\cdot ms^{-1}$]	400000	400000
	K6i [ms^{-1}]	150	150
	K [M^{-1}]	850	850
	R_{max} [ms^{-1}]	10	10
	U_{max} [$M\cdot ms^{-1}$]	2000	2000
	τ_1 [ms]	1	12
	τ_2 [ms]	13	19.9
	φ_1 [mm^{-1}]	0.004	0.0716
	φ_2	0.98	0.7394
	φ_3 [mm^{-1}]	0.0002	0.0305
	φ_4	0.999	0.8889
Module 2	C1i	0.154	0.159
	C1n1	0.01	0.0032
	C1n2	0.15	0.0094
	C1n3	0.01	0.0083
	C1n4 [ms]	85	600
	C2i	0.11	0.109
	C2n1	-0.0315	-0.0435
	C2n2	0.27	0.315
	C2n3	0.015	0.024
	C2n4 [ms]	70	80
	C3 [ms]	54.717	54.717
	C4	-18.847	-18.847
	C5	3.905	3.095
	α	1.65	1.5
Module 3	K_{SE} [mm^{-1}]	0.16	0.35
	$P_{0.5}$ [N]	100.3	17.5
	g_1 [mm]	-0.0045	0.0003
	g_2 [mm]	0.0981	0.1637
	g_3	0.6211	0.4002
	a_0 [N]	0.4	-34.675
	b_0 [$mm\cdot s^{-1}$]	99.7	-130.31
	c_0 [N]	-57.1	-57.86
	d_0 [$mm\cdot s^{-1}$]	42.2	-202.78

Figure 1

Click here to access/download;Figure;fig1.tif

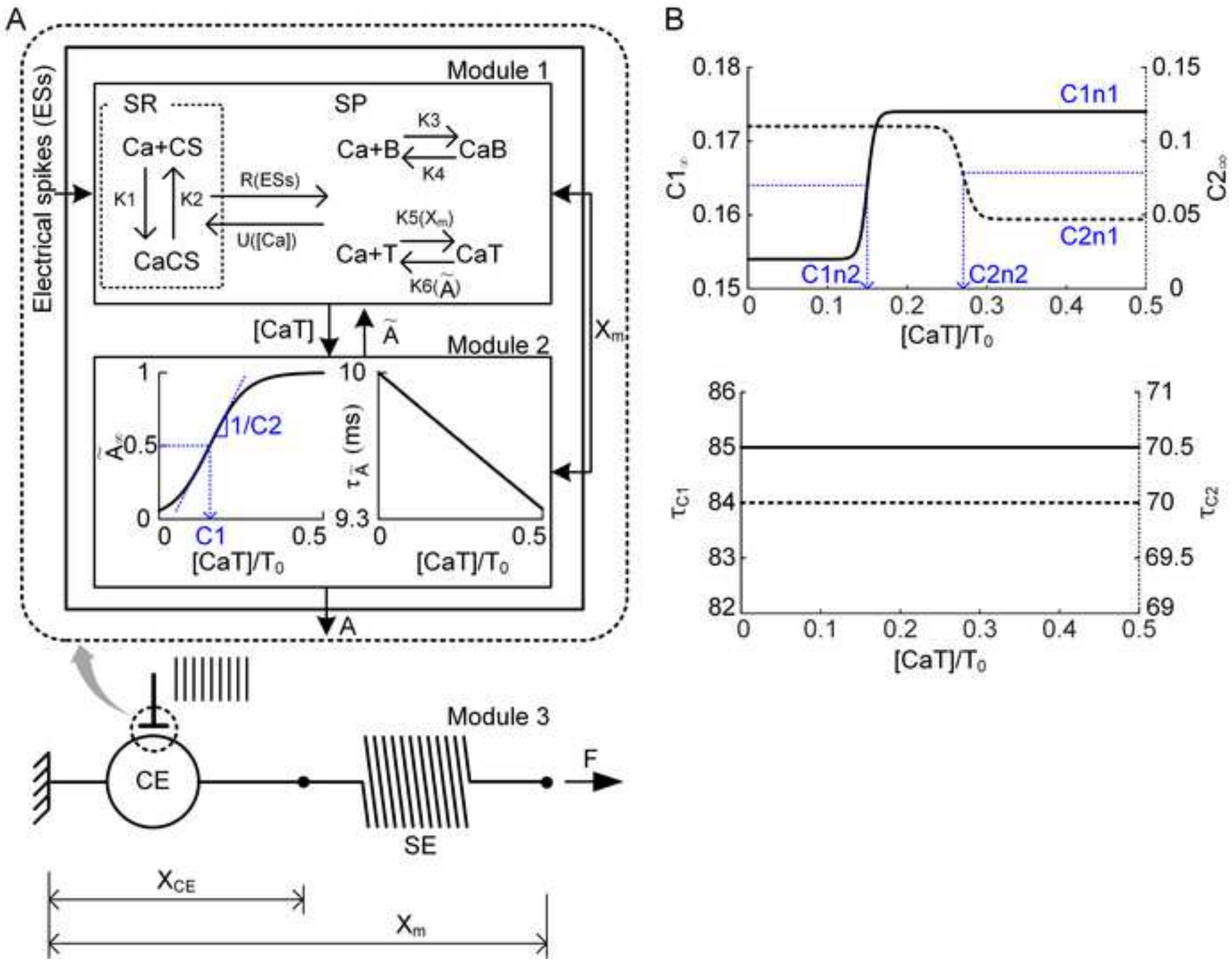

Figure 2

Click here to access/download;Figure;fig2.tif

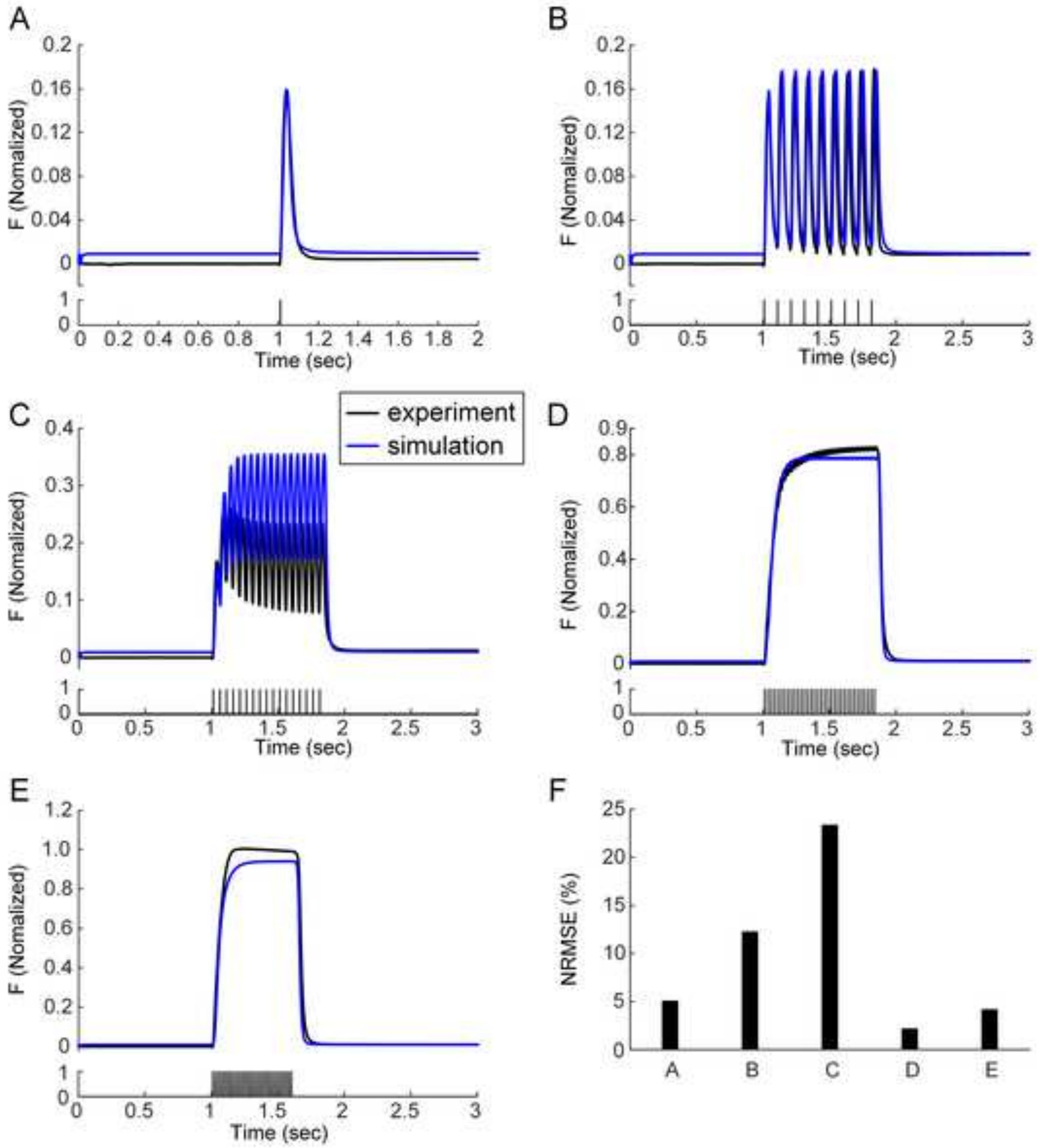

Figure 3

Click here to access/download;Figure;fig3.tif

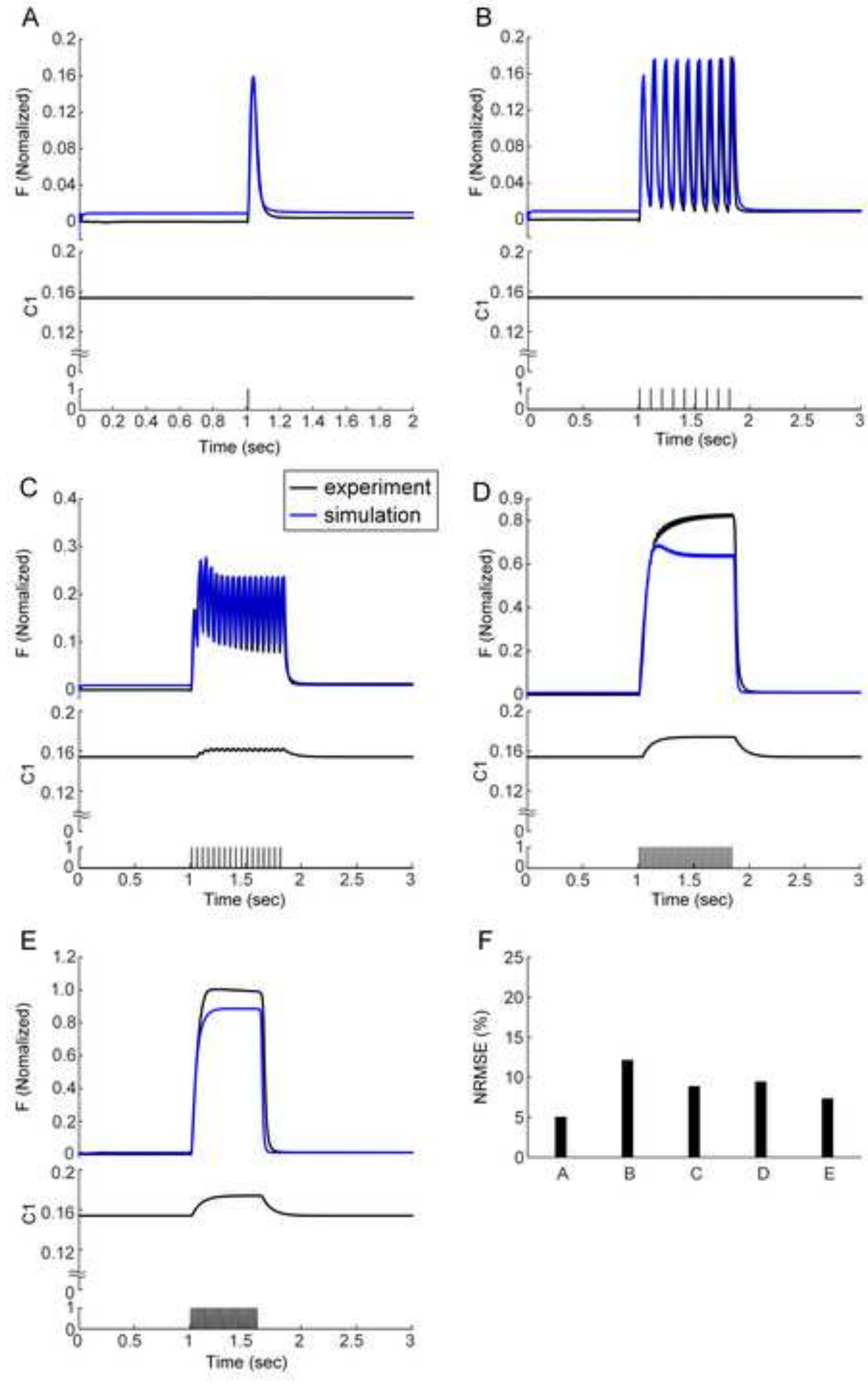

Figure 4

Click here to access/download;Figure;fig4.tif

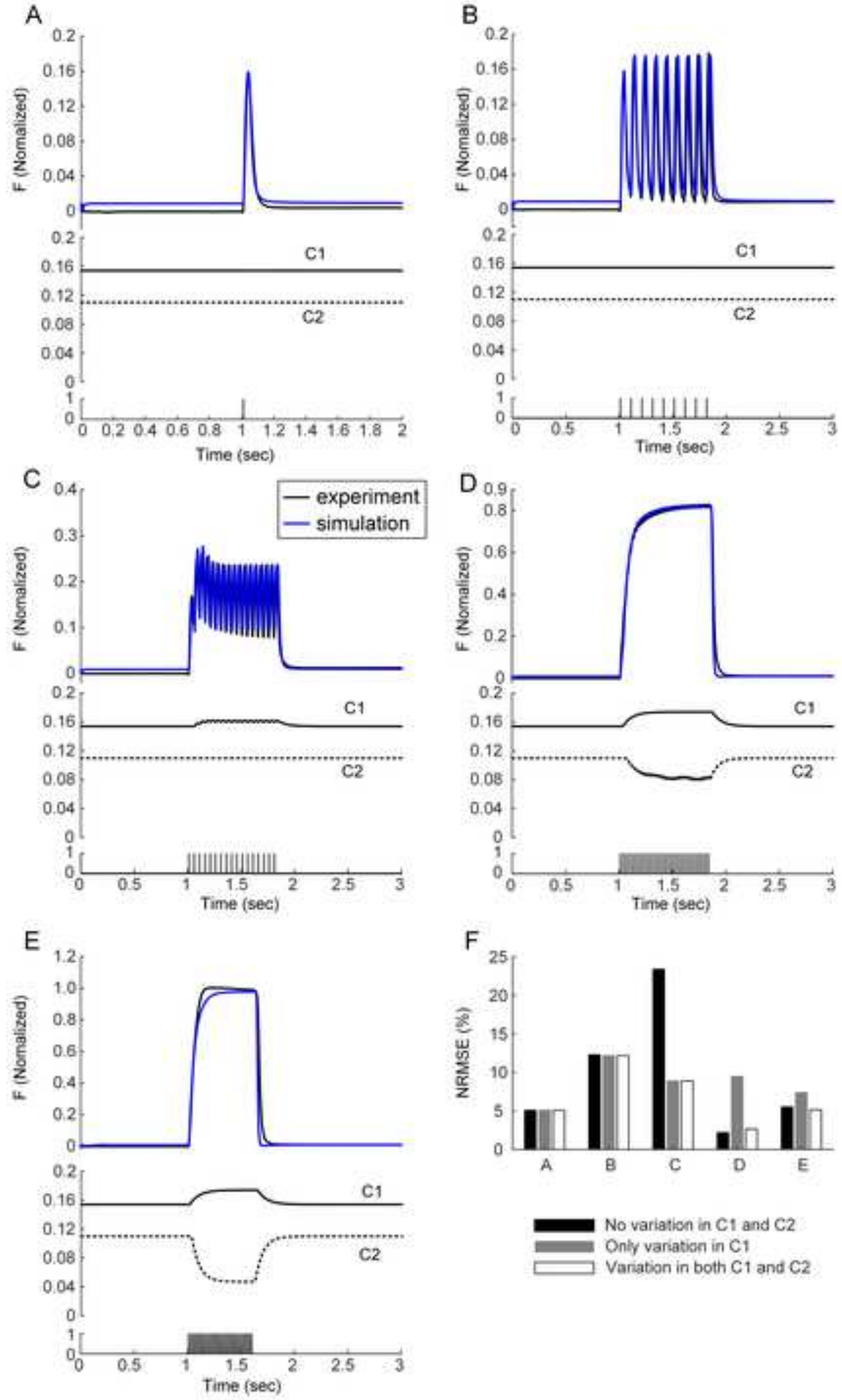

Figure 5

Click here to access/download;Figure;fig5.tif

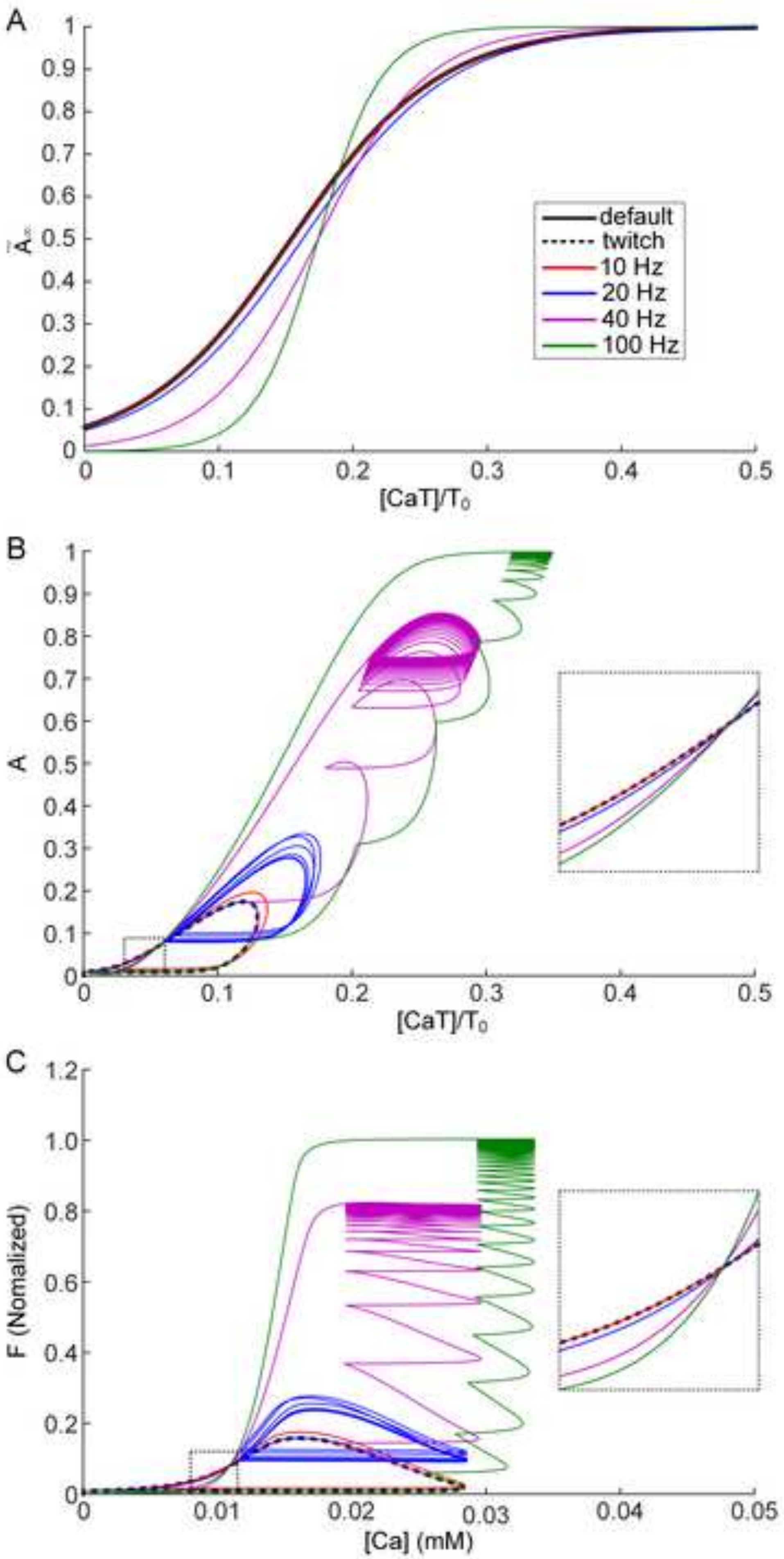

Figure 6 Click here to access/download;Figure;fig6.tif

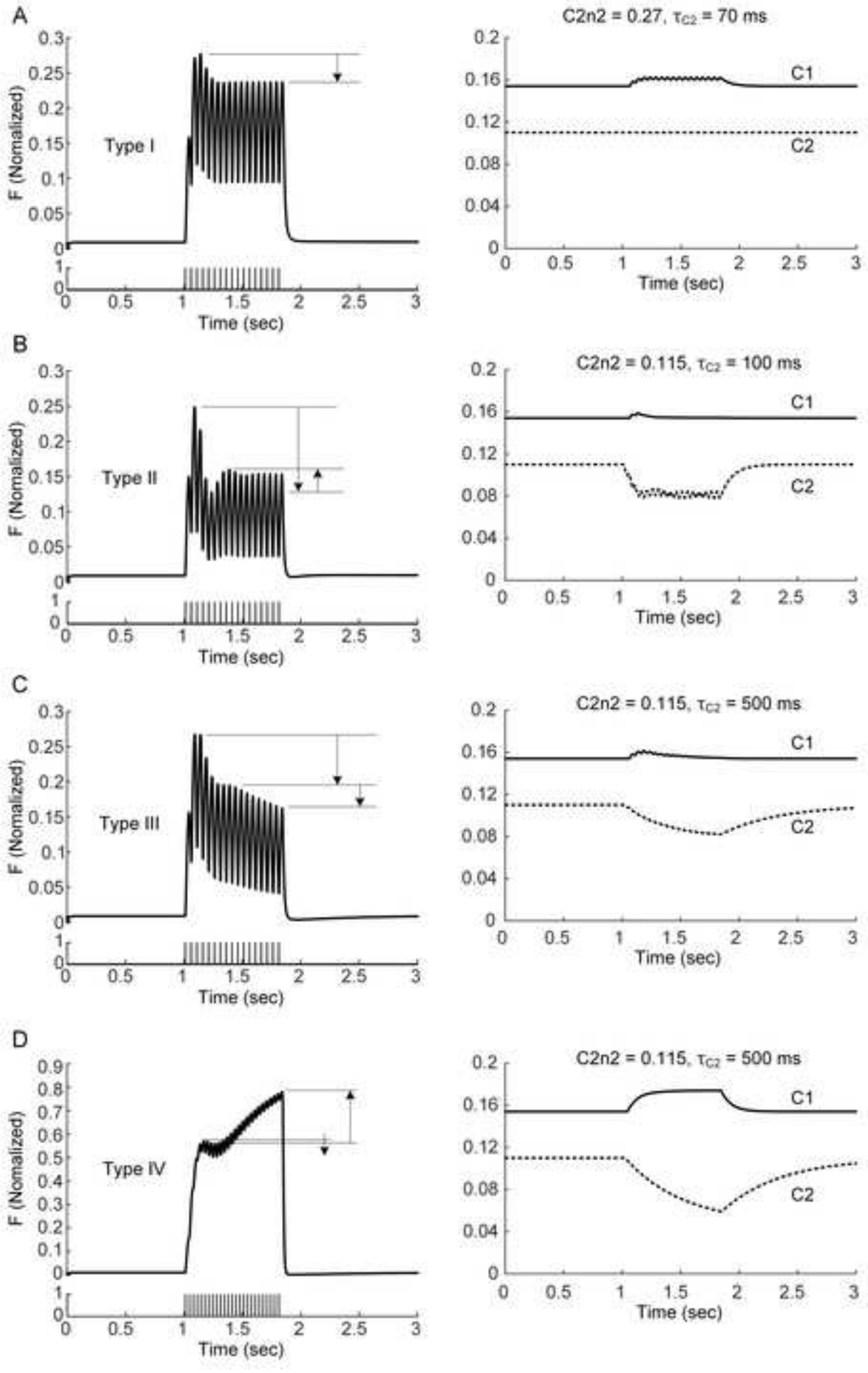

Figure 7

Click here to access/download;Figure;fig7.tif

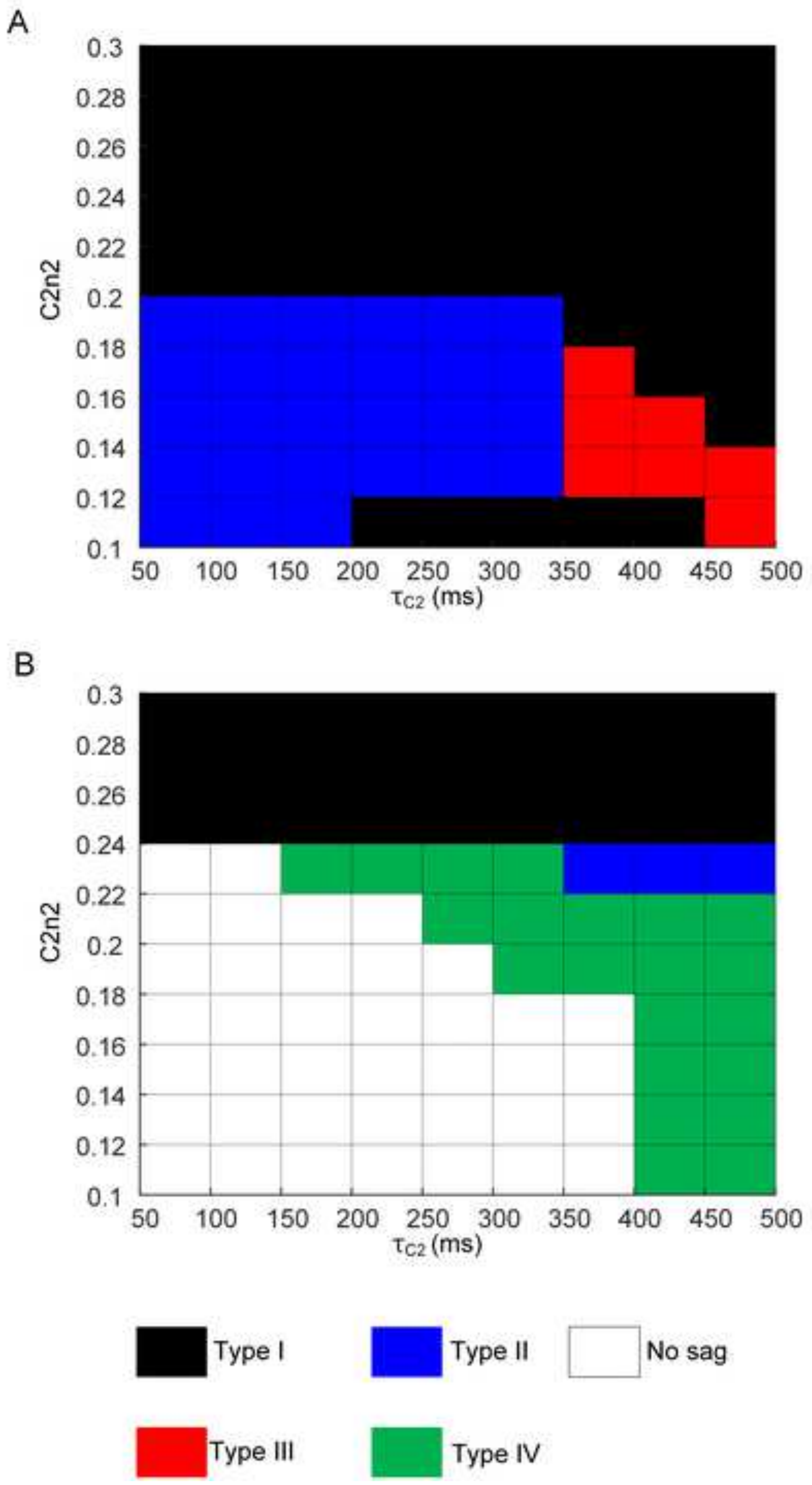

Figure 8

Click here to access/download;Figure;fig8_new.tif

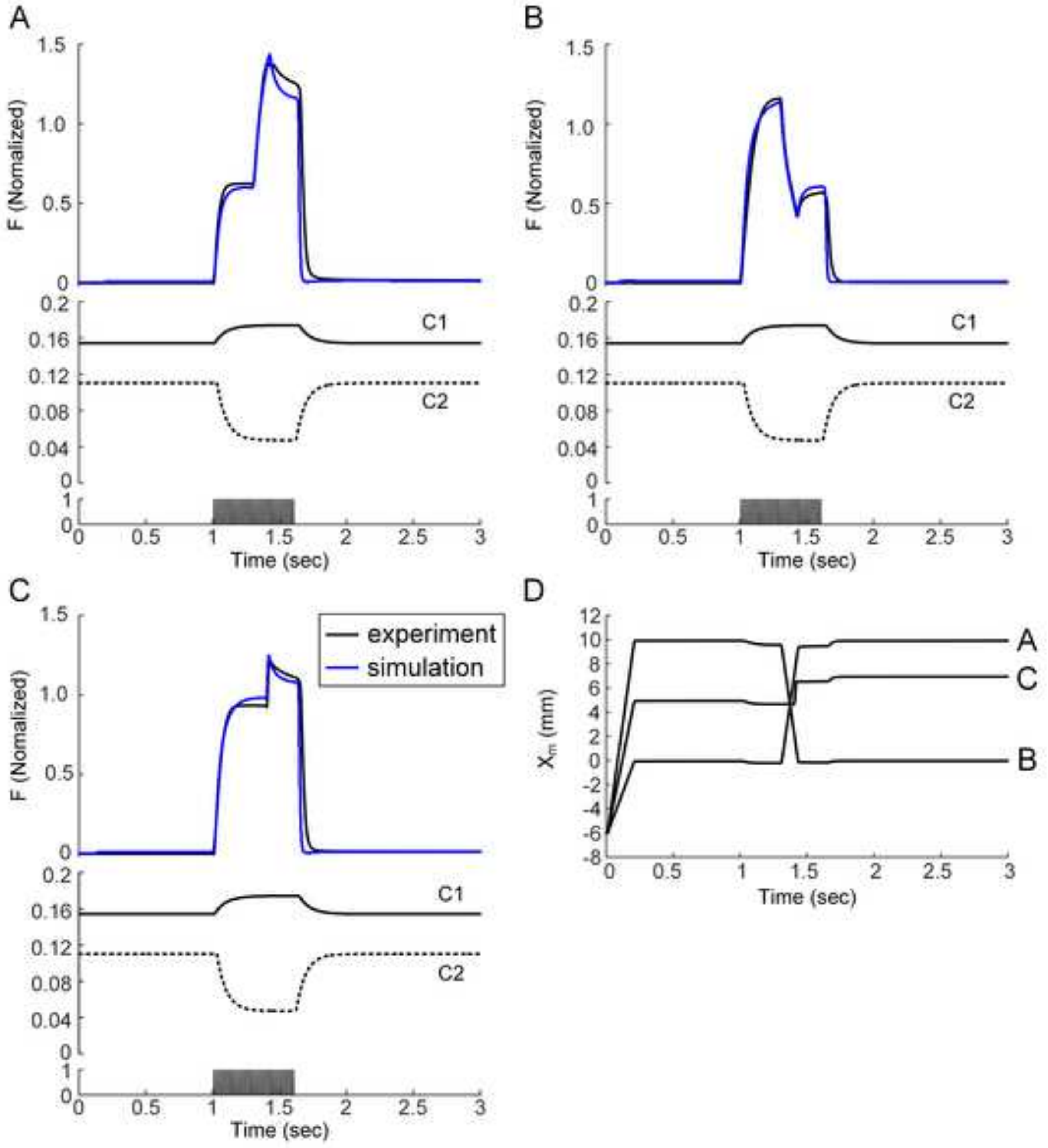

Figure 9

Click here to access/download;Figure;fig9.tif

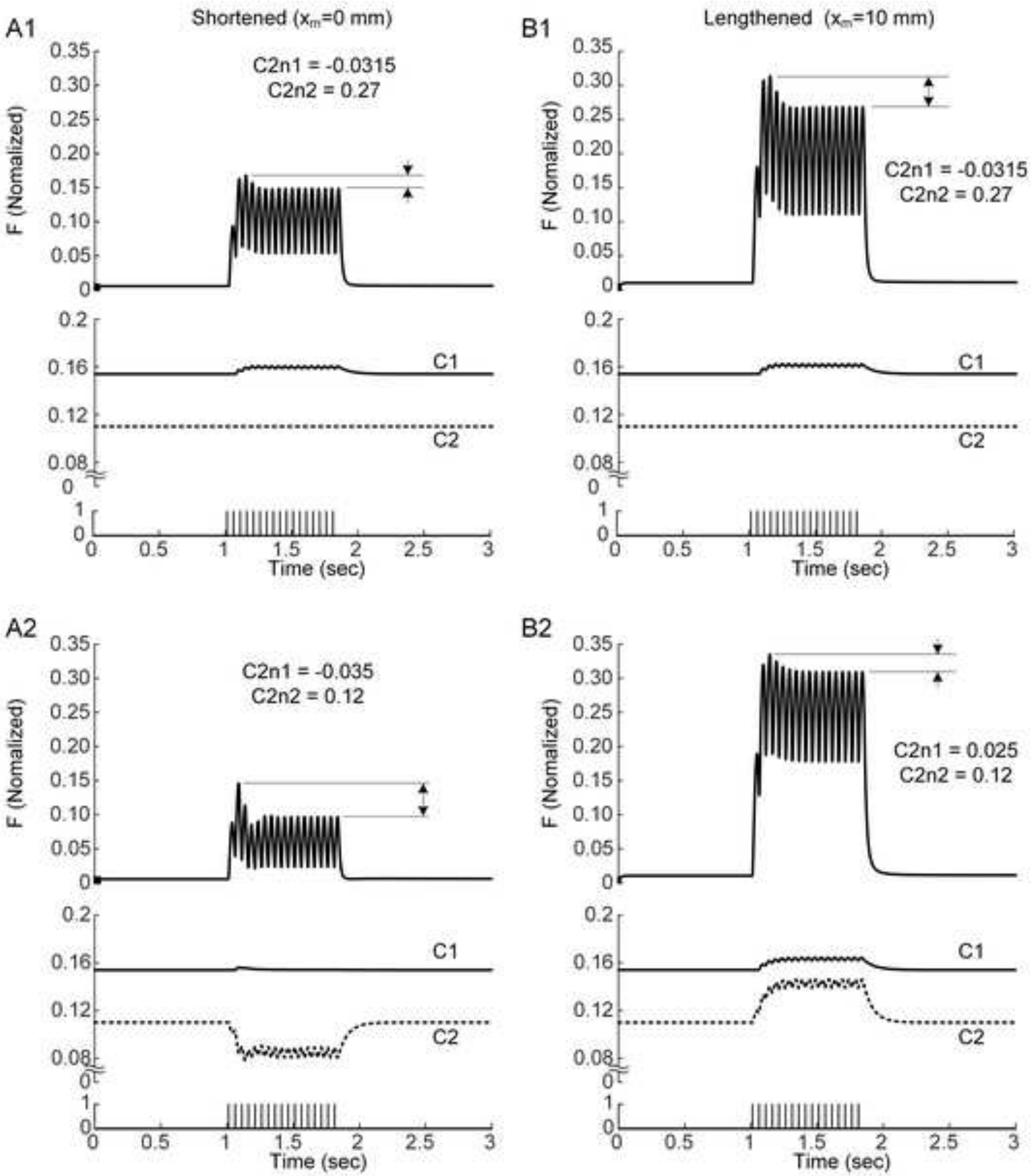

Supplemental Figure S1

Click here to access/download
Supporting Information
fig s1.tif

Supplemental Figure S2

Click here to access/download
Supporting Information
fig s2.tif

Supplemental Figure S3

Click here to access/download
Supporting Information
fig s3.tif

Supplemental Figure S4

Click here to access/download
Supporting Information
fig s4.tif

Supplemental Figure S5

Click here to access/download
Supporting Information
fig s5.tif

Supplemental Figure S6

Click here to access/download
Supporting Information
fig s6.tif

Supplemental Figure S7

Click here to access/download
Supporting Information
fig s7.tif

Supplemental Appendix S1

Click here to access/download
Supporting Information
supplemental appendix s1.docx

Codes for models and simulations

Click here to access/download

Supporting Information - Compressed/ZIP File Archive

supplemental data s1.zip
