# Supplementary material for: A dynamic calcium-force relationship model for sag behavior in fast skeletal muscle
Source: PLoS Comput Biol. 2023 Jun 8;19(6):e1011178. doi: 10.1371/journal.pcbi.1011178 (PMC10284414; doi:10.1371/journal.pcbi.1011178)
Supplement: Attachment — Submitted filename: PLOSOne_reviewer_comments.docx [file pcbi.1011178.s017.docx]

The authors propose a modification to a previously published model of muscle force production due to electrical stimulation. Specifically, they have examined whether calcium sensitivity may, in fact, be a dynamic process that can account for experimental observations seen *in situ*, such as muscle sag, which are not replicated by static calcium sensitivity parameters. Although a somewhat modest modification to an already published model, the manuscript justifies the rationale of adding this feature and provides a compelling argument that its presence improves the model’s correlation to experimental observations. Furthermore, the manuscript explores some of the implications of dynamic calcium sensitivity, including finding a novel sag ‘type’. The model development and rationale is well justified with references to physiological studies and the study seems to be rigorous with findings that could have important implications to understanding muscle force production and control. The author’s conclusions are appropriately conservative and many of the model limitations are acknowledged. Some of my concerns and suggestions are provided below.

Major Comments

Methods

- φ1-φ4 are never mentioned prior to Line 249 and for Module 1 they are the parameters that vary between the two cats (orders of magnitude in the case of φ3). I know it was described in the previous publication of the soleus model, but briefly these parameters should be better described along with their physiological relevance. It says they are “length dependent changes in calcium sensitivity”, but I’m left wondering “Does it makes sense that they would be different between the two cats?” I can’t answer that question with the information currently provided in this manuscript.
- There are several variations on the Hill-type muscle model. Is there any reason this particular one was chosen? If a different variant was chosen, for example one which included the parallel element or dampening, would that effect the results? In Fig 2C. it is shown that the muscle-tendon model without changes in C1 and C2 overestimates force production at 20 Hz compared to experimental data – thus making an argument for an improved model. However, would the increased stiffness due to a parallel element also improve the model’s prediction? Likewise, would the dampening and its velocity-dependency introduce a sag-like behavior? While I suspect the answer to both questions is ‘no’, it would be good to justify this simple hill-type model.

Results

- Lines 310-313: I would argue that Figure 2 shows the inadequacy of the previous model which had no variation in calcium sensitivity instead of showing the “necessity” of variation in calcium sensitivity. We can’t completely exclude the possibility that other modifications to the model could also improve its performance or generate the previously missing sag behavior.
- Lines 418-433: The assertion that “...changes in the midpoint and slope of the calcium-force relation may be crucial for the force-length and force-velocity properties…” should be expanded upon. I’m not convinced. The force-length relationship is commonly attributed to the relative proportion of overlap of the thick and thin filaments, while the force-velocity relationship is thought to arise from the temporal dynamics of the “power-stroke” cycle and the percentage of myosin heads that are phosphorylated vs dephosphorylated at a given instance relative to the frequency of stimulation, neither of which are represented in this model. Obviously, Ca dynamics are critical in these processes, but it might be more accurate to say that the model captures some of those dynamics without directly representing them, but I think even that claim requires a more thorough justification. Furthermore, does the previous model, which does not have variation in calcium sensitivity, not display force-length and force-velocity relationships?

Minor Comments

Typo Line 142: “strain gage-based”

If data processing is to be described using only a reference to a commercial software’s implementation, e.g. the ‘smooth’ function in Matlab or the ‘cnexp’ method in NEURON, it would be good to include the software version information for reproducibility.
