# Supplementary material for: A dynamic calcium-force relationship model for sag behavior in fast skeletal muscle
Source: PLoS Comput Biol. 2023 Jun 8;19(6):e1011178. doi: 10.1371/journal.pcbi.1011178 (PMC10284414; doi:10.1371/journal.pcbi.1011178)
Supplement: Attachment — Submitted filename: ReplyToReviewers__rev1_f.doc [file pcbi.1011178.s019.doc]

April 15, 2023

The Editorial Office

PLOS Computational Biology

Reply to Reviewer Comments

**RE: Revision of the manuscript “A dynamic calcium-force relationship model for sag behavior in fast skeletal muscle”**

Thank you for the opportunity to resubmit the revision of our manuscript and reply to the concerns raised by the Reviewers. We believe the process of review and revision has created a much stronger manuscript that will communicate our novel findings to the readership of PLOS Computational Biology. Our point-by-point responses (Arial in purple) to the Reviewers’ comments (Times New Roman in black) are given below with line numbers. We have taken the Reviewers’ concerns seriously and made significant changes to the original manuscript.

Reviewers' comments:

**Reviewer #1:** The authors propose a modification to a previously published model of muscle force production due to electrical stimulation. Specifically, they have examined whether calcium sensitivity may, in fact, be a dynamic process that can account for experimental observations seen *in situ*, such as muscle sag, which are not replicated by static calcium sensitivity parameters. Although a somewhat modest modification to an already published model, the manuscript justifies the rationale of adding this feature and provides a compelling argument that its presence improves the model’s correlation to experimental observations. Furthermore, the manuscript explores some of the implications of dynamic calcium sensitivity, including finding a novel sag ‘type’. The model development and rationale is well justified with references to physiological studies and the study seems to be rigorous with findings that could have important implications to understanding muscle force production and control. The author’s conclusions are appropriately conservative and many of the model limitations are acknowledged. Some of my concerns and suggestions are provided below.

Reply: We truly appreciate your valuable comments and constructive suggestions.

Major Comments

Methods

- φ1-φ4 are never mentioned prior to Line 249 and for Module 1 they are the parameters that vary between the two cats (orders of magnitude in the case of φ3). I know it was described in the previous publication of the soleus model, but briefly these parameters should be better described along with their physiological relevance. It says they are “length dependent changes in calcium sensitivity”, but I’m left wondering “Does it makes sense that they would be different between the two cats?” I can’t answer that question with the information currently provided in this manuscript.

Reply: We apologize for this confusion. The parameters of φ1-φ4 were determined to match twitch responses at shortened, intermediate, and lengthened muscle-tendon lengths under isometric conditions. In the experiments, twitch amplitude decreased by 4.15% at the shortened length and increased by 7.5% at the lengthened length relative to the intermediate length in CAT14. However, twitch amplitude decreased by 89% at the shortened length and increased by 211% at the lengthened muscle relative to the intermediate length in CAT12. This variation in twitch amplitude caused φ1-φ4 to vary between the two cats. To address this issue, we have added sentences describing the equations involving φ1-φ4 to the Methods in the revised manuscript (Lines 211-215).

- There are several variations on the Hill-type muscle model. Is there any reason this particular one was chosen? If a different variant was chosen, for example one which included the parallel element or dampening, would that effect the results? In Fig 2C. it is shown that the muscle-tendon model without changes in C1 and C2 overestimates force production at 20 Hz compared to experimental data – thus making an argument for an improved model. However, would the increased stiffness due to a parallel element also improve the model’s prediction? Likewise, would the dampening and its velocity-dependency introduce a sag-like behavior? While I suspect the answer to both questions is ‘no’, it would be good to justify this simple hill-type model.

Reply: The Reviewer directs our attention to an important issue. In this study, we employed Hill-type mechanics comprising a contractile element and an elastic element in series because it is simple and effective for modular modeling of the essential processes that the intact muscle undergoes for force production under physiological conditions. In particular, we did not include the parallel passive elements in our muscle model because this study focuses on the active properties of muscle contraction over the physiological range of muscle length and stimulation frequency. Thus, the model parameters were determined to replicate the isolated active force by subtracting the passive force measured without muscle excitation from the total force measured with muscle excitation under the same conditions.

Furthermore, to address the reviewer’s concerns, we tested whether parallel passive elements play a role in inducing sag behavior. In the present study, we adopted the physiological range of muscle length variation from the data obtained from cats during their locomotor-like movements. Over this range of muscle lengths, the passive force was constant and very low compared to the active force (Figure S15 A). In addition, the sag profile including both the active and passive forces was shifted downward in comparison with the profile including only the active force at the same muscle-tendon length (Figure S15 B). This result indicates that the dampening of parallel elements and the velocity dependency of the process may not significantly impact sag behavior.

To alert the readers to the critical issue raised by the reviewer, we have revised the text (Lines 178-181) in the Methods and added new sentences (Lines 754-761) and a supplementary figure (Figure S15) to the Discussion in the revised manuscript.

Results

- Lines 310-313: I would argue that Figure 2 shows the inadequacy of the previous model which had no variation in calcium sensitivity instead of showing the “necessity” of variation in calcium sensitivity. We can’t completely exclude the possibility that other modifications to the model could also improve its performance or generate the previously missing sag behavior.

Reply: We agree with the reviewer. Following the reviewer’s suggestion, we have changed the sentence to “This result indicates the insufficiency of the previous model, with no variation in calcium sensitivity, for predicting force production by fast muscle during unfused isometric contractions.” in the revised manuscript (Lines 404-406).

- Lines 418-433: The assertion that “...changes in the midpoint and slope of the calcium-force relationship may be crucial for the force-length and force-velocity properties…” should be expanded upon. I’m not convinced. The force-length relationship is commonly attributed to the relative proportion of overlap of the thick and thin filaments, while the force-velocity relationship is thought to arise from the temporal dynamics of the “power-stroke” cycle and the percentage of myosin heads that are phosphorylated vs dephosphorylated at a given instance relative to the frequency of stimulation, neither of which are represented in this model. Obviously, Ca dynamics are critical in these processes, but it might be more accurate to say that the model captures some of those dynamics without directly representing them, but I think even that claim requires a more thorough justification. Furthermore, does the previous model, which does not have variation in calcium sensitivity, not display force-length and force-velocity relationships?

Reply: We apologize for the confusion caused by this miscommunication. We agree with the reviewer that multiple factors, including the dynamics of crossbridge interaction, determine the force-length and force-velocity properties. In the present study, the model parameters (i.e., g1-g3 and a0-d0) in Module 3 were forced to reproduce the length-tension (L-T) and velocity-tension (V-T) relationships measured under full excitation with 100 Hz frequency stimulation. From the perspective of sliding filament theory, the values of those parameters reflected the number (N) of force-generating crossbridges formed through spatiotemporal actomyosin interactions underlying the L-T and V-T relationships. However, for partial excitation with low-frequency stimulation, changes in the fraction of N were reflected by the A-CaT relationship of Module 2. In this study, thus, the number of crossbridges in the force generating state at a given time corresponds to the product of the maximal tension produced in a given length and velocity (T(L, V)) and A under physiological input conditions.

The primary purpose of this analysis was to show that the muscle-tendon model with variations in the calcium-force relationship is capable of replicating the L-T and V-T relationships, as seen in the experiment under full excitation. What we found interesting was that the slope steepening of the calcium-force relationship could sufficiently compensate for the depression effect by the rightward drift of the calcium-force relationship for the reproduction of both the L-T and V-T relationships in fast muscle (Figure S3). This result suggests the importance of the net effect of both changes in the midpoint and the slope of the calcium-force relationship in predicting the L-T and V-T relationships for fast muscle. Furthermore, our previous model for slow muscle could display the L-T and V-T relationships with no variation in the calcium-force relationship. This result might indicate the type-specific actin-myosin interactions in slow and fast skeletal muscle as muscles reach the full activation state.

To prevent the readers from being misled, we have added sentences in the Methods (Lines 331-341) and Results (Lines 522-525) along with a supplementary figure (Figure S3) that clearly shows the influence of the slope of the calcium-force relationship on the L-T and V-T relationships in the revised manuscript.

Minor Comments

- Typo Line 142: “strain gage-based”

Reply: The typo has been corrected to “gauge” in the revised manuscript (Line 145).

- If data processing is to be described using only a reference to a commercial software’s implementation, e.g. the ‘smooth’ function in Matlab or the ‘cnexp’ method in NEURON, it would be good to include the software version information for reproducibility.

Reply: We have added the software version information for MATLAB (version R2012a) and NEURON (version 6.1) in the revised manuscript (Line 148 and 359).

**Reviewer #2:** The manuscript entitled “Modular modeling framework reveals dynamic alterations in calcium sensitivity during force production in skeletal muscle” by Kim and Heckman, describes a modification of a previously published modular computational framework, to reproduce the so called “sag behavior” observed in unfused isometric contraction of cats medial gastrocnemius, where fast myosin isoforms are predominant. Because the sag behavior is not observed in slow muscles, the authors propose to modify the previously published modular computational framework designed for cat soleus, where slow myosin isoforms are predominant, introducing a dynamically varying calcium-force relation, specifically introducing a dependence from the actin activation (described by the ratio CaT/T0), in the pCa50 (here called C1) and in the Hill coefficient (here called C2).
With the assumed modification, the model is able to roughly reproduce the force-time behavior at different frequencies optimizing four parameters for each of the two variables, C1 and C2. Based on these evidences the authors deduce several insights about the importance of this mechanism in generating the “sag behavior”, as well as the relative contribution of the two parameters in modulating the sag types.

Reply: We truly appreciate your valuable comments and constructive suggestions.

Major remark:

- My major remark is about the impact of the proposed results. The approach proposed in the manuscript is mainly phenomenological, since a number of parameters are modulated to optimize the fitting of a single set of experimental curves (from cat14). The need of modifying almost all the parameters in module 2 (the most relevant ones) to reach a proper fitting for the second set of data (Cat12), doesn’t lead to a validation of the model itself, but more to a sensitivity analysis.

Reply: We agree with the Reviewer that in the present study the calcium-force relationship experimentally observed in skinned muscle fibers was phenomenologically represented in a mathematical form in module 2 of our model. This modeling approach was chosen due to the lack of experimental data for the molecular mechanisms underlying the calcium-force relationship, and the purpose of this study was to investigate the mesoscopic property of the calcium-force relationship during force production in fast skeletal muscle under physiological conditions. In addition, we do not argue that the model trained with the first dataset (CAT14) can predict the second dataset (CAT12) without modification of the model parameters. Our intention for this analysis was to confirm the applicability of our modular modeling approach for the dataset showing different sag behaviors. To prevent the readers from being misled, we have added the sentence to the Methods (Lines 271-275) and changed the subtitle “Validation of model predictions“ to “Model predictions for a different cat” in the revised manuscript (Line 528).

- Moreover, the model is missing a number of known effects which are influenced by the activation level, force, dynamical behavior etc. Some of them are recognized by the authors as limits, some others are not (for instance the force-driven activation of the thick filament at sub-maximal thin filament activation, Linari et al Nature 2015). These effects will strongly affect the final behavior of the muscle and greatly limit the possibility of deducing the molecular mechanisms behind the sag behavior. I agree with the authors about the importance of phenomenological approaches to simulate muscle behavior at higher scales in multidimensional models, and that the proposed model can represent a computational framework for physiologically realistic model.

Reply: We also agree with the reviewer that multiple factors, including activation, force and activity, may influence the microscopic properties, such as thin and thick filament activation, that affect the final behavior of the muscle. To the best of our knowledge, however, how these microscopic changes impact the mesoscopic properties of the calcium-force relationship remains unknown under physiological conditions. To tackle this issue, we developed a modular modeling framework that allowed us to divide the complex process of force generation into subprocesses that can be separately modeled and validated by relevant experimental data. In this modeling framework, the first module (for calcium dynamics) and the third module (for force production) were constrained by the relevant experimental data. Only the second module (representing the calcium-force relationship) was adjusted for the model to replicate the force data measured under physiological conditions. We conducted this study as a first step to determine the dynamics of the calcium-force relationship for force production under physiological conditions. In the next step, we plan to extend the second module to investigate the possible molecular mechanisms underlying the dynamics of the calcium-force relationship during force production under physiological conditions. For clarification, we have added sentences addressing the important features of our modeling approach to the Methods (Lines 170-172, 292-296 and 782-786) in the revised manuscript.

- However, the manuscript is presented mainly as a way to infer the importance of the dynamic alteration of the calcium sensitivity to explain the sag behavior, see sentences at lines 312 (the necessity), 325 (might decrease), 330 (could not explain), 345 (might be induced), 430 (may be crucial), 471 (was required) and 474 (must be steepened), 500 (we demonstrated), 610 (needed to). In particular I found not appropriate to define Type III sag, which have not been experimentally observed, as a “newly found” type “through the analysis … in this study”. For the previous considerations, I advance doubts of the impact of the proposed implications deduced by the model analysis. A way to solve this issue could be to rewrite the manuscript reducing the results inferred about the role of the proposed phenomenological approach, and stressing more its possible role in properly predicting the physiological implications of the sag behavior.

Reply: We are grateful to the reviewer for directing our attention to an important issue. Following the reviewer’s suggestion, we have revised the manuscript to add specific emphasis on the following:

1) Improvement of prediction for sag behavior of fast skeletal muscle via the dynamic alternation of calcium-force relationship (Lines 404-406, 418-420, 423-424, 438-443, 490-492, 522-525, 560-564, 606-609 and 806-809)

2) Possible mechanisms of the dynamic alternations in the calcium-force relationship (Lines 585-609).

3) Influence of Modules 1 and 3 on the sag behavior (Lines 678-765)

In accordance, we have summarized the inferred results in the Discussion section (Lines 566-583) and changed the title to “A dynamic calcium-force relationship model for sag behavior in fast skeletal muscle” in the revised manuscript.

Other major remarks:

- There are many differences between fast and slow fibers and the modifications of the previously published model, setted for the slow muscle, seem to be inappropriate. Cytosolic buffer (B) in fast skeletal muscle is mainly represented by Parvalbumin, which is virtually absent in slow muscle. The new model simply modify the total amount of “B” but not its kinetics, which I think strongly influence the calcium distribution during the first stimuli. Similarly, Troponin in slow muscle has one binding site, and two in the fast muscle. Also, the values proposed in ref. 37, consider the high and low affinity binding sites, so 0.43 mM, as including the low affinity binding site in the troponin. Authors should refer to [www.jgp.org/cgi/doi/10.1085/jgp.201210773](http://www.jgp.org/cgi/doi/10.1085/jgp.201210773) for a better description of the differences between fast and slow models parameters.

Reply: The Reviewer brings up a critical issue. In Module 1, we employed a canonical model to transform electrical stimulation into the concentration of calcium bound to troponin. This model assumes that calcium ions rapidly occupy the regulatory sites of troponin and that the total troponin concentration is similar in both slow- and fast-twitch muscle fibers (Baylor and Hollingworth 2012). However, as the reviewer pointed out, the type-specific properties, such as the predominance of parvalbumin and two troponin regulatory sites in fast muscle fibers, might play a role in determining the sag phenomenon. To clarify this issue, we first evaluated the influence of parvalbumin with no variation in C1 and C2 (C1n1 = C2n1 = 0). The simulations in Figure 2 were repeated with the same kinetic scheme (K3 = 4.17*10^7^ M^-1^*ms^-1^ and K4 = 0.5 ms^-1^) and total concentration (B_0_ = 7.5*10^-4^ M) as reported in a previous study (Baylor and Hollingworth 2012). The results showed a substantial reduction (~ 40% on average) in predicted peak forces, particularly for low stimulation frequencies (< 40 Hz), without inducing sag behavior (Supplemental Figure S9). Then, we assessed the influence of two regulatory sites of troponin. The reaction equation of Ca + T = CaT was changed to 2Ca + T = Ca_2_T in Module 1 to explicitly express the interaction between free calcium ions and the two troponin regulatory sites (Figure 1 A). Accordingly, the system equations (3) and (5) were updated as follows,

The total troponin concentration (T_0_ = 1.2*10^-4^ M) was set to be the same as that used in a previous study (Baylor and Hollingworth 2012). The twitch response was matched to the force data measured at X_m,0.5_ by increasing the calcium release ([Ca_SR_]_init_ = 0.04 M, R_max_ = 139 ms^-1^ and τ_2_ = 14.5 ms) from the SR to compensate for the increase in parvalbumin and troponin site concentration. The reaction constants of K5i and K6i were reset to 1.37*10^6^ M^-2^*ms^-1^ and 0.56 ms^-1^ so that the calcium concentration for half activation (Ca_50_ = sqrt(K6i/K5i) = 6.4*10^-4^ M) became 70% larger and the calcium-force relationship slope steeper than the canonical case (Ca_50_ = K6i/K5i = 3.75*10^-4^ M). The degree of Ca_50_ increase was determined as the average increase in Ca_50_ in fast muscle fibers compared to slow muscle fibers across rat (100% increase in Stephenson et al., 1981 J Physiol), monkey (35% increase in Fitts et al., 1985 J Appl Physiol), and human (80% increase in Ruff 1989 Muscle Nerve) skeletal muscle. As a result, the new model with fast-type-specific properties could not reproduce the sag behavior without variations in C1 and C2 and produced larger forces at high stimulation frequencies (> 20 Hz) due to the steeper calcium-force relationship (Supplemental Figure S10). However, with the C1i (0.162), C2i (0.134), C2n1 (-0.0095), and C2n4 (200 ms) in Module 2 modestly adjusted, the new fast muscle model could reproduce all isometric and isokinetic force data showing dynamic variations in C1 and C2 similar to those for the canonical case (Supplemental Figures S11 and S12).

These results further support the hypothesis that both calcium sensitivity and cooperativity of crossbridge formation dynamically change to produce the sag behavior observed in fast skeletal muscles under physiological conditions. To alert the readers to this important issue raised by the Reviewer, we have added new sentences to the Discussion section along with new supplementary figures in the revised manuscript (Lines 678-720). For review purposes, we have also included the codes for simulations of the fast-type-specific model (Supplemental Figures S11 and S12) as other information in the revised manuscript.

- The model is based on the assumption that the calcium binding and unbinding to troponin is a function of muscle-tendon length and muscle activation level, which affect the force-time behavior at different frequencies, but a detailed analysis of the role of these parameters, relative the variations of C1 and C2, is missing. It is important to discriminate the two components in a more quantitative way.

Reply: The Reviewer is correct. In this study, the calcium binding (K5) and unbinding (K6) to troponin were represented as a function of muscle-tendon length (X_m_) and muscle activation level () in Module 1. The isometric forces in Figure 2 were simulated at shortened (X_m,0_) and lengthened (X_m,1_) lengths with and without the length dependence of K5 (K5i for all X_m_) under no variation in C1 and C2 (Supplemental Figure 13). We also simulated the isometric forces at the intermediate length (X_m,0.5_) with and without the activation dependence of K6 (K6i for all) without C1 and C2 variation (Supplemental Figure S14). The length dependence of K5 had a modest impact on force predictions only for low stimulation frequencies (< 40 Hz) at the shortened state without inducing the sag behavior. However, the lack of activation dependence in K6 caused a considerable reduction (~ 85 ± 0.08%) in the predicted peak forces for all stimulation frequencies without producing sag behavior. These results also support the hypothesis that both calcium sensitivity and cooperativity of crossbridge formation dynamically change to produce the sag behavior observed in fast skeletal muscles under physiological conditions.

To alert the readers to this critical issue raised by the Reviewer, we have added new sentences to the Discussion section in the revised manuscript (Lines 722-734).

- I found the description of the model hard to follow, especially for the need of moving back and forth from the main text to the appendix. I suggest to introduce an extended description of the three modules, with the major equations, in the materials and methods section in the main text.

Reply: Following the Reviewer’s suggestion, we have extended our description of the three modules, including the major equations in the main text of the revised manuscript (Lines 189-267).

Minor:

- Line 133: L4 and S2 are not defined

Reply: We have revised the phrase to “only ipsilateral dorsal roots of the common peroneal nerve (L4‒S2)“ in the revised manuscript (Line 134-135).

- Line 155: Xm should be defined here instead of line 181.

Reply: X_m_ has been defined at the place the reviewer suggested in the revised manuscript (Line 158).

- Line 263: the index “i” is not used

Reply: The equation has been updated in the revised manuscript (Line 353) as follows:

where the subscripts m and s and N indicate measurement, simulation, and the number of data points (indexed with i) considered for evaluation, respectively.

- Legends in figures 2, 3, 4, 8 are in panels C, I suggest to move them in the first panel or in every panel.

Reply: Following the reviewer’s suggestion, we have moved legends in the first panel in Figures 2, 3, 4, and 8 in the revised manuscript.

- The definition of the equation for the uptake of calcium (line 17 appendix) seems not familiar to me, a reference or an explanation could help the reader.

Reply: The equation for the calcium uptake (U) used in a previous study (Westerblad J. Physiology 1994) was adapted for this study considering the absence of an inhibitor (i.e., TBQ) of the pumping action of the sarcoplasmic reticulum in physiological conditions. For clarification, we have added a sentence to the Methods section of the revised manuscript (Lines 202-203).

**Reviewer #3:** I would like to commend you on the development of this model and the modular approach that you have used. I feel that by creating a module model different aspects and physiological parameters that may lead to SAG can be explored. This type of model also may allow researchers to understand particular elements of SAG and the relative contribution of different proteins, ions, structures and general conditions.

Reply: We truly appreciate your valuable comments and constructive suggestions.

Introduction

- Line 102: This has also been demonstrated experimentally by Smith, I. C., Bellissimo, C., Herzog, W., Tupling, A. R., Can inorganic phosphate explain sag during unfused tetanic sag during unfused tetanic contractions of skeletal muscle? Physiol Rep, 4(22), 2016, e13043, doi:10.14814/phy2.13043.

Reply: The reviewer is correct. We have revised the text in the Introduction of the revised manuscript (Line 104).

- Line 105: It might be a good idea to express to the readers how each of these conditions would change the calcium sensitivity relationship so that later when you make these changes in your model, these can be related to potential physiological pathways.

Reply: We appreciate the reviewer bringing our attention to an important issue. In the present study, we focus on the dynamic variation in the calcium-force relationship during the rapid decline in isometric force (within half a second) after its peak under unfused stimulation conditions in fast skeletal muscles. We did not include the decrease in free calcium caused by reduced calcium release from the sarcoplasmic reticulum and accelerated calcium uptake into the sarcoplasmic reticulum. The main reason was that the decline in free calcium under these conditions is known to be a much slower process (on the order of seconds) than the crossbridge formation changes (on the order of milliseconds). To prevent the readers from being misled, we have clearly stated in the Introduction that this study focuses on the dynamic variation in the calcium-force relationship during the rapid force decline in fast skeletal muscles before the final phase of muscle fatigue (Lines 112-114).

Materials and methods

- Line 181: You may also consider adding the fact that calcium also binds to titin in the sarcomere, and this seems to change the elastic properties, and potentially the active force production (Herzog et al., 2018).

Reply: We agree with the reviewer that titin-myofilament interactions may influence active force production during isometric contractions. The present study did not explicitly consider titin-myofilament interactions for predicting active force production during isometric contractions. Instead, extracellular (tendon and aponeurosis) and intracellular (myofilament and titin protein) elastic properties were collectively reflected in the serial elastic stiffness (K_se_) in Module 3. In the present study, K_se_ was constant but could vary to some extent due to changes in titin stiffness upon muscle activation. All simulations in Figures 4 and 8 were repeated over a range of K_se_ values (0.13~0.23 mm^-1^) adopted from a previous study measuring short-range stiffness as a function of force for cat medial gastrocnemius muscles (Cui et al., 2008 J Biomech). The variation in K_se_ over the physiological range had little impact on the prediction of forces during isometric and isokinetic contractions (not shown). Notably, titin-myofilament interactions do not affect the length-dependent calcium activation and force-velocity properties (Kissa Physiology 2020).

To address this issue, we have added corresponding sentences to the Methods (Line 236-237) and Discussion (Line 736-748) in the revised manuscript.

- Line 189: The hill type mechanics as proposed do not explain the activity dependent response of the muscle (force enhancement and force suppression). Or potentially the action / involvement of titin in the serial elastic element. This can be simplified to the hill model but should be explained why it is removed (simplified) and what the effect might be.

Reply: As the reviewer pointed out, titin has also been suggested to be an active spring underlying the activity-dependent response of the muscle, such as residual force enhancement or suppression after active stretching or shortening. We think that this issue is beyond the scope of the present study, which focuses on active force production under isometric and isokinetic conditions. To prevent the readers from being misled, we have clearly addressed this limitation of the current model in the Discussion of the revised manuscript (Lines 748-752).

Discussion

- Line 469: As this is the discussion it might be useful if the authors described how / physiologically / they would suggest this action to be occurring. Reduced force production during the myosin actin interaction, changes in successful binding, increased actin myosin cycling…

Reply: We are grateful to the reviewer for directing our attention to an important issue. As the reviewer mentioned, multiple mechanisms might achieve dynamic alternations in calcium sensitivity. That is, the rightward drift of the calcium-force relationship might be caused by a gradual reduction in the number of force-generating crossbridges and the force per force-generating crossbridge under low pH or high inorganic phosphate concentration (Fitts JApplPhysiol 2008) or in the duration of strong myosin-actin binding during crossbridge cycling. The upward drift of the slope of the calcium-force relationship might be induced by a progressive increase in the transition rate from non-force-generating to force-generating crossbridge formation through myosin light chain phosphorylation (Stull PNAS 1990) or in the binding affinity of myosin heads to actin filaments due to mechanosensitive binding proteins (Luo Biophysical J 2012). We think that the modular modeling framework developed for this study may be easily extended to provide a testbed for the relative contributions of these mechanisms to the calcium-force relationship under physiological conditions. To clarify this issue, we have added corresponding sentences to the Discussion of the revised manuscript (Lines 585-609).

- Line586: It would be useful again to relate changes in model behavior to changes in physiological action, manipulating parameters of the curve which would simulate reduced actin-myosin interaction…(hypothetically).

Reply: We appreciate the reviewer’s suggestion. The static calcium-force relationship was analyzed to infer the calcium dependence of force-producing crossbridge formation under in vitro steady-state conditions. Thus, our study may provide insights into the dynamic aspects of the calcium sensitivity and cooperativity of crossbridge formation under physiological conditions. That is, the calcium sensitivity and cooperativity of crossbridge formation might tend to be constant under low levels of muscle excitation (i.e., < 10 Hz) (Figure 2). However, the calcium sensitivity of crossbridge formation might be progressively lowered, resulting in sag behavior under intermediate levels of muscle excitation (i.e., approximately 20 Hz) (Figure 3). The cooperativity of crossbridge formation might be progressively increased, resulting in force enhancement under high levels of muscle excitation (i.e., > 40 Hz) during isometric and isokinetic contractions (Figures 4 and 8). Furthermore, the cooperativity of crossbridge formation might play a crucial role in determining the form of sag behavior (Figures 6 and 7). All these findings suggest that the calcium sensitivity and cooperativity of crossbridge formation might operationally vary depending on both neural excitation and muscle length in fast muscle under physiological conditions. We have revised the corresponding sentences in the Discussion (Lines 566-583 and 781-782) of the revised manuscript for clarification.
