# Supplementary material for: A dynamic calcium-force relationship model for sag behavior in fast skeletal muscle
Source: PLoS Comput Biol. 2023 Jun 8;19(6):e1011178. doi: 10.1371/journal.pcbi.1011178 (PMC10284414; doi:10.1371/journal.pcbi.1011178)
Supplement: Attachment — Submitted filename: PCOMPBIOL0D22-01287-R1.docx [file pcbi.1011178.s020.docx]

Upon reviewing the revised manuscript, I note that the authors have addressed my previous concerns, updating the manuscript with respect to the suggestions offered by myself and the other reviewers. At present, I find no additional areas requiring extensive further revision.

While the manuscript is technically compelling, it would benefit from a stronger correlation between the model's behavior and its underlying molecular and physiological principles. This deeper connection would provide a more comprehensive understanding of the model's real-world relevance in fast-twitch muscle physiology. However, I acknowledge that such an expansion may be beyond the intended scope of the current paper.

Although this paper lacks a degree of some physiologically relatable interpretations to substantiate the model's operation, it should not hinder its publication. The paper's primary contributions are of significant value, offering a novel perspective that is likely to prompt further discussions and investigations in the field.

I commend the authors for their conscientious efforts to address the issues and questions raised during the review process. Their dedication to refining the manuscript is evident and appreciated.
